# Imperfect detection biases occupancy estimates in plants: A review and case study

**DOI:** 10.1002/ecy.70525

**Published:** 2026-09-23

**Authors:** Anna W. Wyngaarden, Megan L. DeMarche

**Affiliations:** ^1^ Department of Plant Biology University of Georgia Athens Georgia USA

**Keywords:** cryptic, detection probability, granite outcrops, occupancy model, patch occupancy, plant survey, rare species

## Abstract

The distributions of rare species are often estimated from presence–absence data, but undetected occurrences (imperfect detection) can lead to consequential underestimates of occupancy. Despite its standard use in animal ecology, detection probability (*p*) is rarely incorporated into plant occupancy estimates. Plants with cryptic life stages or traits (e.g., seed banks, dormancy) may be especially prone to periods of unobservability, resulting in imperfect detection even when a species is present. We tested the hypothesis that cryptic traits lower detection by reviewing published estimates of *p* in plant systems. While taxa with cryptic traits, particularly annual plants with seed banks and deciduous geophytes, exhibited lower *p*, there was wide variation across genera and publications. We then conducted a case study of the rare, annual, aquatic plant, *Gratiola amphiantha* (snorkelwort), endemic to ephemerally rain‐filled granite rock outcrop pools, to estimate occupancy (Ψ), *p*, and their environmental drivers. We found that Ψ increased with deeper soils and larger pool areas. Snorkelwort exhibited a high but imperfect *p* (mean *p* = 0.87) that varied with competing vegetation cover and census date. Modeling Ψ across varying levels of survey effort showed that ignoring imperfect detection can underestimate total occupancy estimates by up to 43%; however, our empirical *p* estimates indicate a minimum of just two surveys can reach 95% detection for snorkelwort. Our findings demonstrate that imperfect detection is an important and underappreciated limitation in plant occupancy studies, even for species with high detection probability. For rare plants, explicitly incorporating taxon‐specific *p* estimates and marginally increasing survey effort can substantially improve the accuracy of occupancy models.

## INTRODUCTION

A species' area of occupancy is one of the main determinants of its rarity status (Crisfield et al., 2024). Typically, these occupancy estimates rely on field surveys of a species' presence or absence (Kéry, 2011). However, species are rarely detected perfectly even when they are truly present (i.e., imperfect detection), leading to underestimates of distributions (Bennett et al., 2024; Guillera‐Arroita et al., 2014). This is especially consequential for rare species where even a few false absences can significantly distort their perceived rarity status (Emry et al., 2011). While accounting for detection probability is standard for animal systems (Goldstein et al., 2024), it remains largely overlooked in plant ecology, despite clear evidence that plants are not detected perfectly (Chen et al., 2013; Perret et al., 2023). Indeed, a 2014 review of abundance and distribution studies found only 1.4% of the plant studies account for imperfect detection (Kellner & Swihart, 2014).

**FIGURE 1:**
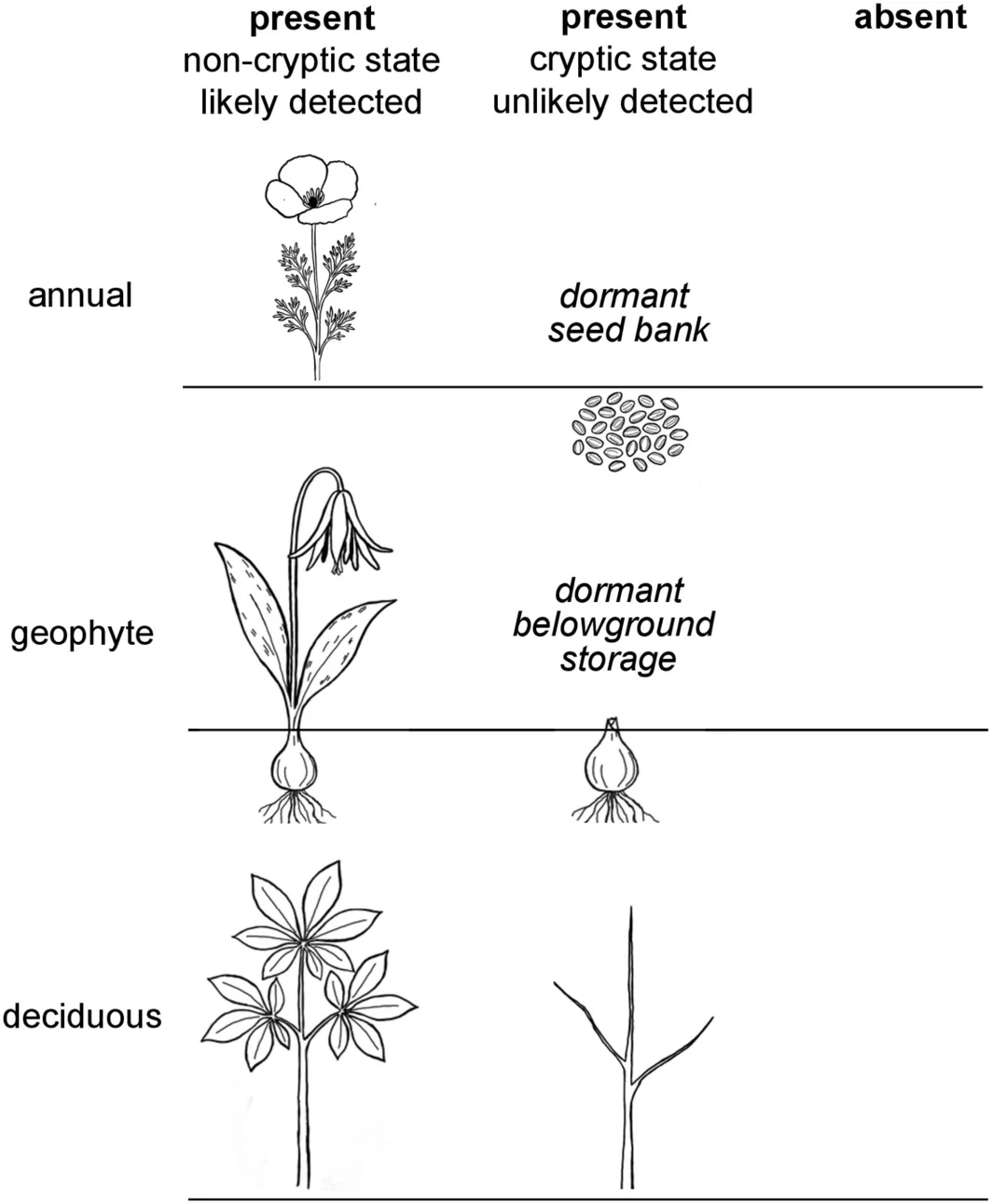
Potential for false absences in taxa with cryptic traits (annual therophytes, deciduous geophytes, and deciduous non‐geophytes). Detectability may be lower during cryptic states (dormant seed bank, belowground storage, or deciduous) compared to non‐cryptic states. Illustrations created by Anna W. Wyngaarden using Procreate (S. I. P. Ltd., 2025).

The probability of detecting a species when it is truly present, detection probability, can be influenced by survey logistics, like surveyor experience (Garrard et al., 2013; Hauser et al., 2022), or time spent observing (Garrard et al., 2008; Perret et al., 2023). But detectability can also heavily depend on a species' ecology, like its morphological characteristics (Chen et al., 2013), herbivory pressure (Alexander et al., 2009), population density (Dennett & Nielsen, 2018), or the immediate surrounding vegetation (Hauser et al., 2022). Cryptic traits, such as annual plants with seed banks (Louvet et al., 2021), perennial geophytes with belowground dormancy (Lauriault & Wiersma, 2019), or deciduousness, likely reduce detection probability by making plants undetectable (dormant belowground) or highly inconspicuous (deciduous without foliage) for portions of their lifecycle (Figure 1). These traits represent a gradient of putative crypsis: Annuals' unobservable seed banks and brief aboveground presence are likely the most cryptic, whereas deciduous species are likely least cryptic because they remain visible, though harder to identify, when dormant. When detection drivers are ignored, seasonal fluctuations in visibility can be easily misconstrued as changes in true habitat occupancy. While some studies have accounted for detection probability precisely because a plant is known to be cryptic (Alexander et al., 2009), the effect of cryptic traits on detection has not been reviewed.

Here, we combine a literature review and case study to address the effect of imperfect detection in plants on inferences of species' occupancy. First, we reviewed the literature on detection probabilities (*p*) in plant systems uniquely focusing on how cryptic traits (annual seed banks, deciduous geophytes, and deciduous non‐geophytes) influence detection. Second, we used repeat surveys of an annual plant, *Gratiola amphiantha* (syn. *Amphianthus pusillus*) D. Estes & R.L. Small (Plantaginaceae), to evaluate its detection probability (*p*), identify the habitat characteristics influencing its occupancy (Ψ) and *p*, and test how accounting for *p* alters Ψ estimates. We then estimate the minimum number of surveys needed within a season to achieve 95% confidence that truly occupied pools are detected present in at least one survey (Olea & Mateo‐Tomás, 2011). Together, this work illustrates the importance of incorporating detection probabilities into occupancy estimates for species of conservation concern.

## METHODS

### Literature review

We conducted a literature search on ISI's Web of Science on June 26, 2024 for publications between the year 2000 and the search date, capturing the period immediately preceding the emergence of modern occupancy models incorporating imperfect detection (MacKenzie et al., 2002). Using the Topic keywords “occupancy” OR “occupancy model” OR “occupancy modeling,” and “plants” OR “floristic,” and “detectability” OR “imperfect detection” OR “detection,” returned 85 publications. We screened these for those that (1) focused on natural populations of plants or lichens, (2) involved observations in the field (this excluded studies empirically testing *p* using simulations or decoys), and (3) directly reported detection probabilities of taxa. Many publications mentioned repeating surveys in their methods but did not report the probability of detection based on these repeat surveys, excluding them from the review. Estimates of *p* came from occupancy models that directly model detection probability as a function of covariates (see Wyngaarden, A.W. 2026 for full literature review details). Only 16 publications met our criteria, from which we compiled *p* estimates for 1427 taxa, averaging across covariates or surveys when multiple estimates were provided.

We then classified each taxon based on three traits‐life cycle (annual or perennial), life form (based on Raunkiaer's system; Adamson, 1939), and deciduousness (deciduous or evergreen). We obtained trait data for each taxon using the Global Inventory of Floras and Traits (GIFT) database (GIFT package; Weigelt et al., 2019) using the traits, “Lifecycle,” “Life form 2,” and “Deciduousness” in R (version 4.2.3; R Core Team, 2023). If trait data from the *GIFT* database were incomplete or conflicting, we determined species traits from additional sources which include other public databases.

We grouped the 1427 taxa into four categories based on their type of crypsis, a gradient representing how often a plant is dormant or unobservable: annual therophytes (*N* = 237), deciduous geophytes (*N* = 81), deciduous non‐geophytic perennials (*N* = 401), or non‐cryptic (*N* = 708). We considered annual therophytes, deciduous geophytes, and deciduous non‐geophytic perennials as putatively cryptic.

We modeled *p* from our literature review using generalized mixed models with a beta distribution in the R package, glmmTMB (version 1.1.10; Brooks et al., 2017). We used the Smithson and Verkuilen (2006) transformation,

$$y=\frac{p\;\left(N-1\right)+0.5}{N},$$

where *N* is the total sample size, to accommodate zeros in the dataset. We fitted three separate mixed‐effects models to evaluate factors influencing *p*. First, we compared cryptic versus non‐cryptic taxa (cryptic state). Second, we compared if cryptic type influences *p*, then tested for significant differences among cryptic types with an estimated marginal means post hoc test with multiple comparison adjustments (emmeans package; Lenth & Piaskowski, 2026). Third, we compared deciduous versus evergreen taxa (deciduousness), fitting a model for perennial taxa only. We evaluated the significance of fixed effects with Wald χ^2^ tests. We included random intercepts for genus and publication in all models to account for taxonomic and study variation. To quantify how much variation in *p* was due to crypsis, we partitioned the variance of all three models into fixed effects, publication and genus random effects, and residual variance (Schulz et al., 2025), using the insight package (Lüdecke et al., 2019). All analyses were conducted in R.

### Case study

**FIGURE 2:**
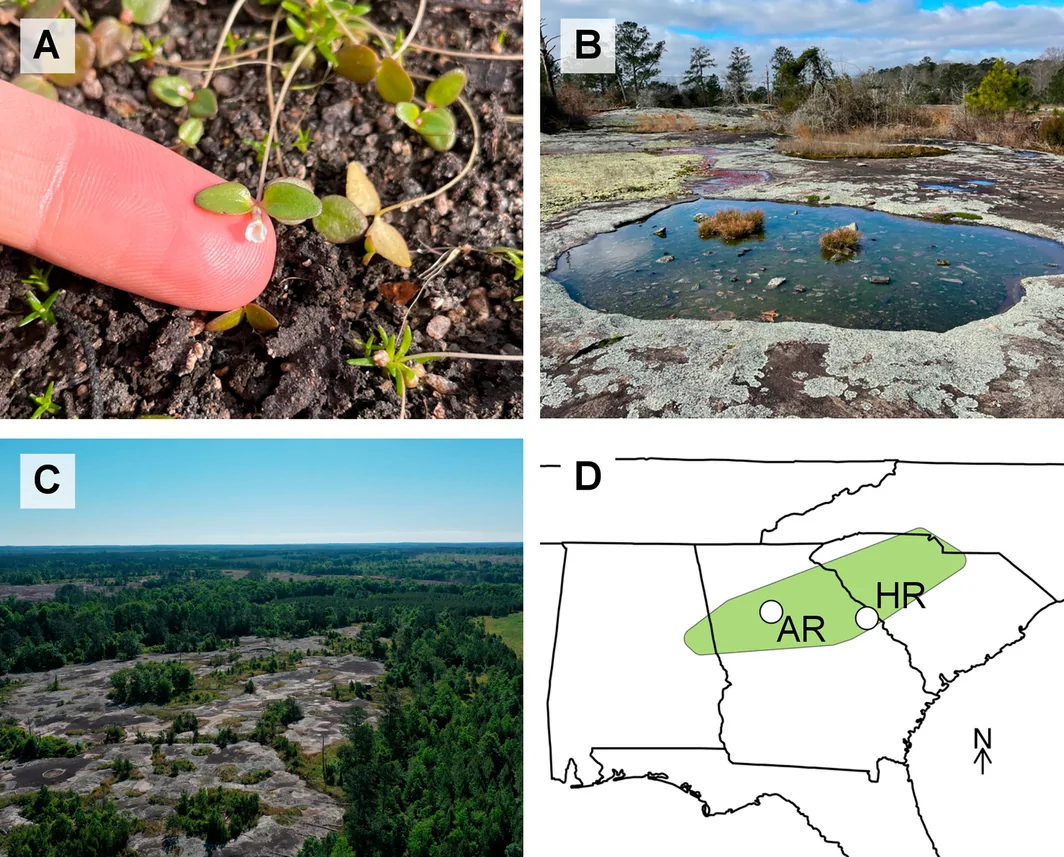
Study system and distribution. (A) *Gratiola amphiantha* in a dry granite outcrop pool, (B) rain‐filled pool, (C) granite outcrops across the landscape, and (D) species range (green) with study sites (dots). Images in panels (A)–(C) were taken by the author, Anna W. Wyngaarden.

We conducted a case study to estimate detection probability (*p*) and occupancy probability (Ψ) for *G. amphiantha* (snorkelwort), a rare, aquatic annual endemic to rain‐filled granite rock outcrop pools in the Southeastern United States (Figure 2; Frings & Davenport, 2017). Snorkelwort can complete its life cycle in 3–4 weeks (Lunsford, 1939), and maintains a dormant seed bank (A. Wyngaarden, unpublished data; Baskin & Baskin, 1978). Pools vary significantly in area, soil depth, and competing vegetation cover, leading to drastic seasonal fluctuations in hydrology. Rapid pool drying may decrease both the true occupancy state and the detectability of the species. To account for this, we explicitly modeled pool characteristics as drivers of *p* and Ψ. Snorkelwort is listed as a federally threatened species under the United States Endangered Species Act; therefore, all field work and research was conducted pursuant to required permits with landowner permissions.

We conducted repeat surveys for snorkelwort at two sites in Georgia: Arabia Mountain (AR) and Heggie's Rock (HR). In February 2022, we mapped all pools containing snorkelwort in each study area (located in the central portion of each outcrop; Appendix S1: Figure S1), as well as unoccupied pools within 5 m of an occupied pool that met suitable habitat criteria (AR: *N* = 217, HR: *N* = 90). The average distance between occupied pools was 5 m in 2022, representing a reasonable seed dispersal range. We included unoccupied pools to account for undetected snorkelwort, dormant seed banks, or colonizations between years.

From 2023 to 2025, one experienced observer conducted 2–4 repeated surveys during each growing season (December–March), recording the species' visual presence (1) or absence (0) in each pool (9 surveys total per site; Appendix S1: Table S2). We recorded three pool characteristics that may influence *p* and Ψ: (1) pool area derived from mapped polygons, (2) soil depth taken at six random points per pool in 0.5‐mm increments, and (3) estimated percent cover of heterospecific vegetation in 2023. All characteristics were treated as pool constants. Soil depth does not change significantly over short timescales (Burbanck & Phillips, 1983), and we confirmed that vegetation cover does not change significantly within the study period by comparing 2023 field estimates with pool images taken in 2022.

### Occupancy models

We fit single‐season occupancy models to each year of our detection data to evaluate how site, habitat covariates, and day of survey influenced Ψ and *p* in each year (Appendix S1: Table S3). Occupancy models account for imperfect detection by estimating *p* from repeat surveys (MacKenzie et al., 2002). We fit separate models to each year because the true occupancy state of a pool can change between years due to colonization or extinction (A. Wyngaarden, unpublished data). We considered site, pool area (area), average soil depth (soil), or competitive vegetation cover (veg) as predictors of Ψ or *p* and also considered day of survey (doy) as a predictor of *p*. Our candidate model set included all possible combinations of predictors, excluding any model that used identical predictors for both Ψ and *p*, as well as models with both parameters as constants, resulting in 162 models (Table 1). We defined doy relative to a November 1 start date and divided this value by 10 for model convergence. We ran models using the occMod function in the RPresence package (MacKenzie et al., 2002) and ranked them based on Akaike information criterion (AIC) weights (Akaike, 1974). Models including doy for 2023 and 2024 did not converge because these datasets have too few repeat detections (3 and 2, respectively). We thus excluded models with doy from the set of candidate models, resulting in a subset of 81 candidate models for 2023 and 2024.

**TABLE 1:** Summary of the top‐ranked models for estimating occupancy and detectability for snorkelwort each year.

Year	Model	∆AIC	*w*	*N*_par_	−2ll
2023	Ψ (area + soil), *p*(veg + site)	0.00	0.219	6	778.51
	Ψ (area + veg + soil), *p*(site)	0.181	0.200	6	778.69
	Ψ (soil), *p*(veg + site)	1.383	0.110	5	781.89
	Ψ (area + veg + soil), *p*(1)	1.863	0.086	6	780.37
	Ψ (1), *p*(1)	28.415	0.000	2	814.92
2024	Ψ (area + soil + site), *p*(veg)	0.00	0.132	6	553.036
	Ψ (soil + site), *p*(area + veg)	0.093	0.126	6	553.129
	Ψ (soil + site), *p*(veg)	0.130	0.124	5	555.166
	Ψ (area + soil), *p*(veg + site)	0.369	0.110	6	553.405
	Ψ (soil), *p*(veg + site)	0.627	0.096	5	555.663
	Ψ (soil), *p*(area + veg + site)	1.395	0.066	6	554.431
	Ψ (area + soil + site), *p*(1)	1.772	0.054	5	556.808
	Ψ (soil + site), *p*(1)	1.955	0.050	4	558.991
	Ψ (1), *p*(1)	34.164	0.000	2	595.200
2025	Ψ (area + soil + site), *p*(1)	0.00	0.242	5	1020.09
	Ψ (area + soil + site), *p*(veg)	0.286	0.210	6	1018.37
	Ψ (area + soil + site), *p*(doy)	0.660	0.174	6	1018.75
	Ψ (area + soil + site), *p*(veg + doy)	0.965	0.149	7	1017.05
	Ψ (area + veg + soil + site), *p*(1)	1.977	0.090	6	1020.06
	Ψ (1), *p*(1)	36.683	0.000	2	1062.77

Abbreviations: *N*_par_, number of parameters in the model; *p*, detection probability; *w*, Akaike information criterion model weight; ∆AIC, change in Akaike information criterion value from the top model; −2ll, twice the negative log‐likelihood; Ψ, occupancy probability.

For each year, multiple candidate models fell within ∆AIC ≤2 of the top‐ranked models, so we averaged models by their AIC weight (*w*) to estimate Ψ and *p*. While we acknowledge the statistical challenges associated with model‐averaging regression coefficients when predictors are collinear (Banner & Higgs, 2017; Cade, 2015), our covariates exhibited low collinearity (all *r* ≤ 0.45; Appendix S1: Figure S2), resulting in highly stable parameter estimates across model combinations (Appendix S1: Table S5). Furthermore, model‐averaged estimates were qualitatively consistent with the top‐supported models (Appendix S1: Figure S4).

We hypothesized that pools with larger area and deeper soil would improve water‐retention capacity, increasing Ψ (Beissinger et al., 2022). Conversely, we expect larger area to reduce *p* via increased survey area (Tucker et al., 2021). Alternatively, larger pools may have higher population abundance, which could increase *p*. Deeper soils may increase *p* by delaying drought‐induced senescence. Finally, we expected vegetative cover would reduce resource availability for Ψ and obscure visual detection for *p*. However, *p* may increase with vegetative cover if it is indicative of a pool's' ability to better retain water. Detailed hypotheses and justifications for each covariate are provided in Appendix S1: Table S1.

### Effects of survey effort on estimates of occupancy

We next evaluated the impact of imperfect detection on estimates of Ψ, and the potential for additional surveys to mitigate this effect. We calculated the minimum number of surveys, *N*_min_, needed to achieve a 95% probability of detecting a truly occupied pool in at least one survey, following Olea and Mateo‐Tomás (2011), as follows:

$$N_{\min}=\frac{\log\left(0.05\right)}{\log\left(1-p\right)},$$

where *p* is the model‐averaged estimate of *p* for each pool.

## RESULTS

### Detectability varies across taxa and is influenced by cryptic state

**FIGURE 3:**
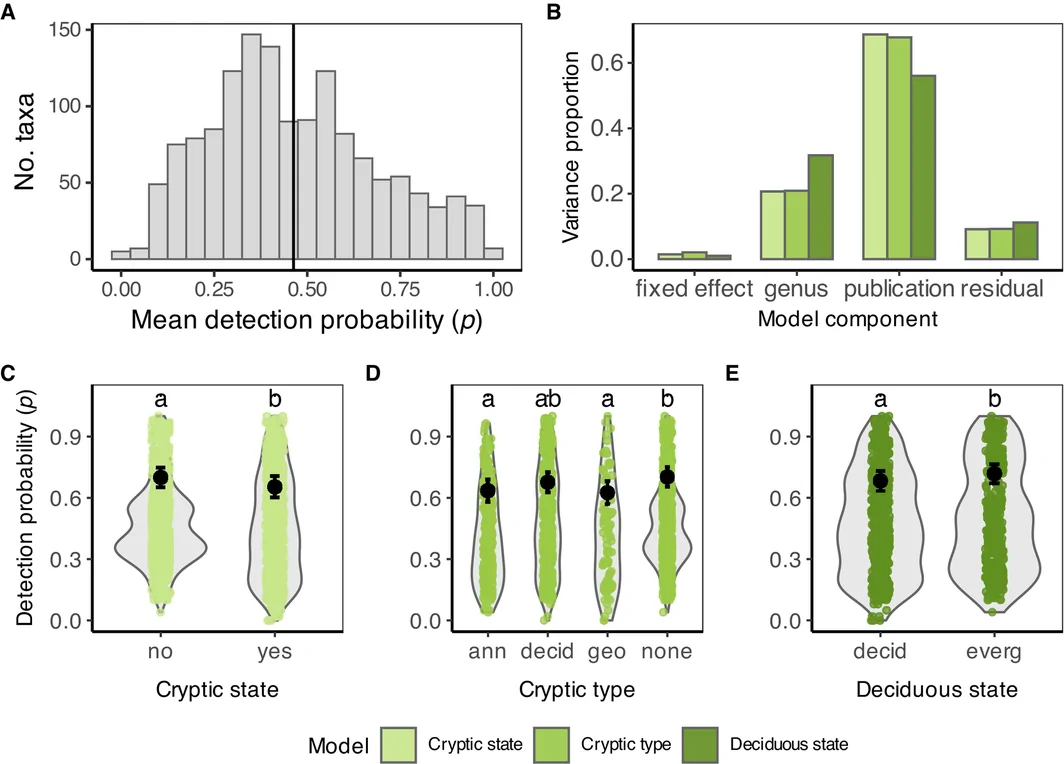
Literature review results for detection probability (*p*). (A) Frequency of reported *p* values (*N* = 1427; mean *p* = 0.46, dark line). (B) Variance partition for mixed models; fixed effects correspond to panels (C)–(E). (C) *p* is lower for taxa with cryptic states (yes) than without cryptic states (no). (D) Cryptic state type (annual [ann], deciduous [decid], geophyte [geo], none) affects *p*. (E) For perennials, *p* is lower for deciduous (decid) than evergreen (everg) taxa. Points and error bars represent model‐estimated means ± 95% CIs; letters denote significant differences. Shades of green correspond to the fixed effect of each model.

Of the cryptic taxa (50.4% of total taxa), 33% were annual therophytes, 11% were deciduous geophytes, and 56% were deciduous non‐geophytic perennials. Separately, we categorized perennial taxa (*N* = 808) by foliage type; of these, 60% were deciduous (*N* = 482) and 40% were evergreen (*N* = 326). Detection probabilities (*p*) were highly variable across taxa with a mean of 0.46 (SD = 0.22; Figure 3A). For all three models testing the effects of cryptic states on *p*, most of the variance was attributed to the random effects of genus and publication, with cryptic traits only explaining between 1% and 2% of the variance (Figure 3B; Appendix S1: Table S1). Yet, cryptic taxa still had significantly lower *p* compared to noncryptic taxa overall (χ^2^ = 12.613, df = 1, *p*‐value = 0.0004; Figure 3C; Appendix S1: Table S1). The type of cryptic trait had a significant effect on *p* (χ^2^ = 20.63, df = 3, *p*‐value = 0.0001). Post hoc Tukey‐adjusted comparisons showed that both geophytes (β = −0.346 ± 0.110, *z* = −3.158, *p*‐value = 0.009) and annuals (β = −0.305 ± 0.077, *z* = −3.934, *p‐*value = 0.0005) had significantly lower *p* than noncryptic taxa, whereas deciduous perennials did not (β = −0.121 ± 0.067, *z* = −1.805, *p‐*value = 0.271; Figure 3D). There were no significant differences among the three cryptic groups. Separately, deciduous taxa had significantly lower *p* than evergreen perennials (χ^2^ = 5.905, df = 1, *p‐*value = 0.0151; Figure 3E).

### Snorkelwort detectability is influenced by competitive vegetation cover and site

**FIGURE 4:**
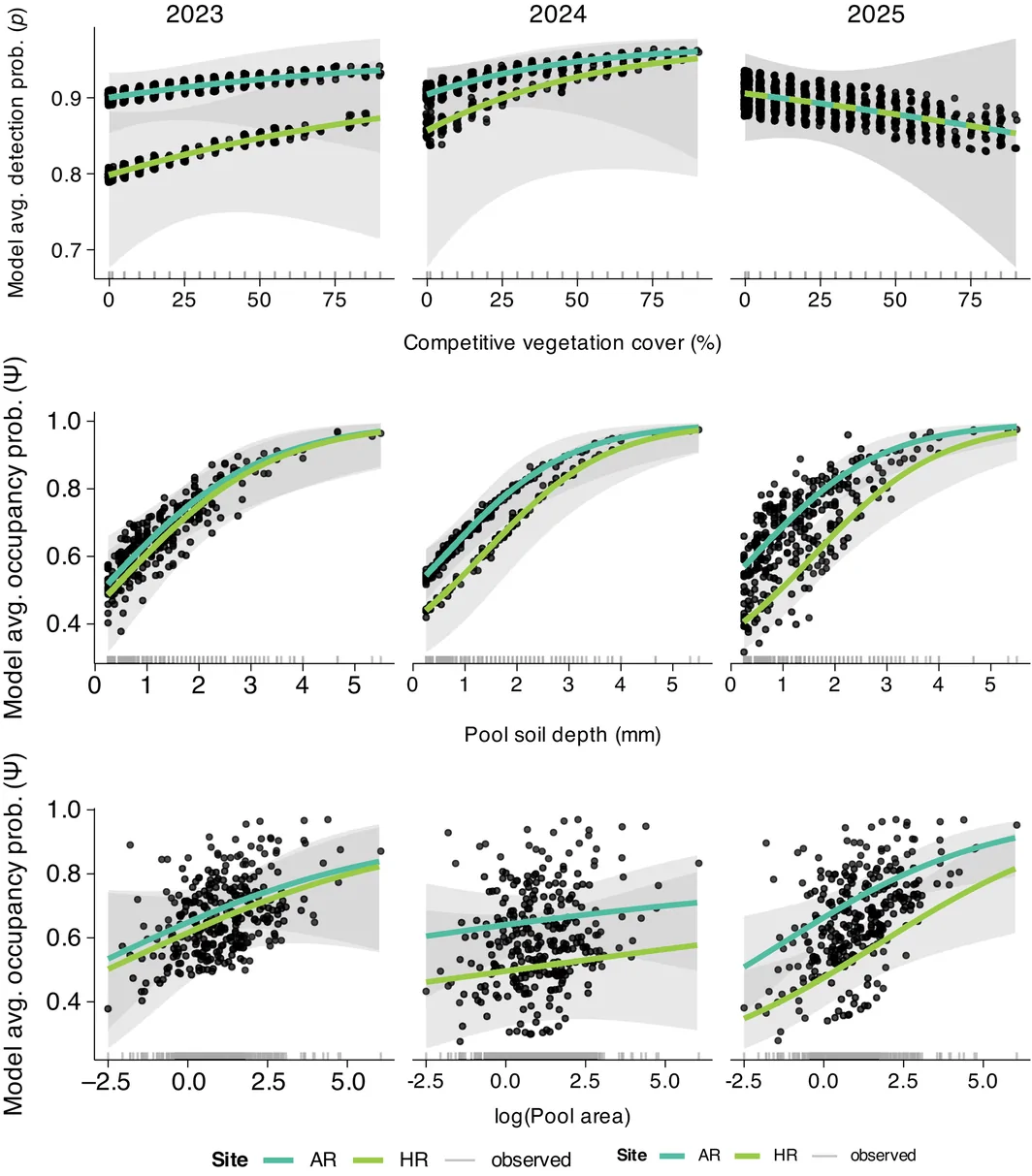
Detection (*p*) and occupancy (Ψ) probabilities vary across years and between sites. Detection probability varies with vegetation cover while occupancy probability increases with deeper soil and larger pools. Solid lines represent model‐averaged estimates (difference in Akaike information criterion between models [∆AIC] ≤ 2) with remaining predictors held constant. Lines are colored by site (Arabia [AR], teal; Heggie's Rock [HR], green), and shaded regions indicate 95% CIs. Points represent model‐averaged pool estimates.

In our case study, 4.6%–28.6% of pools were detected to have snorkelwort present in at least one, but not all, surveys in a single season (Appendix S1: Figure S3). Competitive vegetation strongly predicted *p* across years, appearing in the best fit model for 2023 and 2024 and in models within ∆AIC ≤2 of the best fit model for all years (Table 1). Vegetation increases *p* in 2023 and 2024 but decreases *p* in 2025 (Figure 4). Site was a top predictor for *p* in 2023 and 2024 and is generally higher at AR than HR. Pool area and survey day of year were weakly supported, appearing in models within ∆AIC ≤2 of the best fit model in 2024 and 2025, respectively. Model‐averaged *p* decreased with pool area in 2024 and later surveys in 2025 (Appendix S1: Table S5).

### Snorkelwort Ψ is strongly driven by deeper soils, larger pool area, and site

We found strong and consistent support that occupancy probability (Ψ) is driven by soil depth, pool area, and site, with these three predictors in the best fit model in all 3 years (Table 1). Occupancy (Ψ) was highest in deeper soils (3–5 mm), larger pools, and in the AR site (Figure 4; Appendix S1: Table S5). We found some support for vegetative cover as a predictor of Ψ, appearing in models within ∆AIC ≤2 of the best fit model in 2023 and 2025. However, vegetative cover had inconsistent effects on Ψ, increasing it in 2023 and decreasing it in 2025. Model‐averaged estimates of Ψ ranged from 0.13 to 0.97 across pools and years (Figure 4).

### Two repeat surveys are sufficient to reach 95% probability of snorkelwort detection

Snorkelwort *p* is high with an overall average of 0.87 (Appendix S1: Figure S5). Estimates of *p* for specific pools ranged from 0.798 to 0.977 across years. Using these values, estimates of *N*_min_ ranged from 0.80 to 1.87 with an overall average of 1.46 (Appendix S1: Figure S6). Thus, two repeat surveys within a season are sufficient to ensure 95% certainty of detection in truly occupied pools.

### Single survey designs underestimate snorkelwort occupancy

**FIGURE 5:**
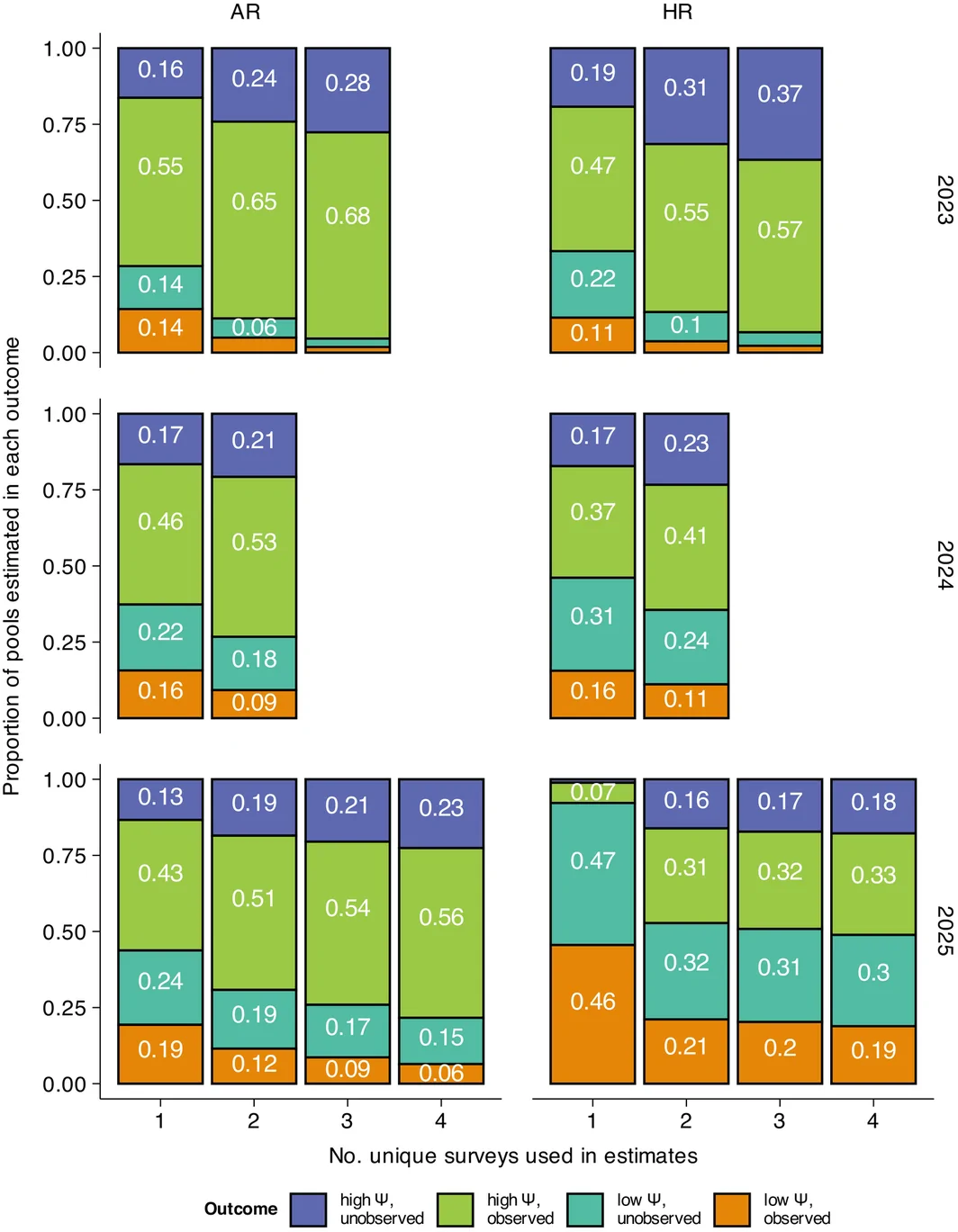
Survey effort affects occupancy‐observation outcomes by site and year. Stacked bars show proportions of pools categorized by their occupancy‐observation outcome for each year and site (Outcome 1 [purple]; Outcome 2 [green]; Outcome 3 [teal]; Outcome 4 [orange]). High Ψ ≥ 0.50 and low Ψ < 0.50. AR, Arabia; HR, Heggie's Rock.

We found strong effects of survey effort on model estimates of Ψ and *p* (Appendix S1: Table S6; Figures S7 and S8). Low survey effort, particularly single survey designs that fail to consider *p*, result in estimates of Ψ that substantially underestimate true occupancy (Figure 5). Specifically, single survey models were much more prone to two types of errors: a higher proportion of pools estimated to have a low Ψ that were in fact observed present (Outcome 4) and a lower proportion of pools classified with a high Ψ that were observed present (Outcome 2). Additional surveys increased the proportion of pools estimated to have a high Ψ (≥0.50), such that more pools that were observed absent over the season were classified with higher Ψ (Outcome 1) and fewer were classified with lower Ψ (Outcome 3), indicating stronger effects of habitat covariates and modeled *p* on predictions of potentially suitable pools (Figure 5; Appendix S1: Figure S8). The effects of survey effort on Ψ were most prominent in the 2025 dataset; a single survey estimated high Ψ at just 56% of pools at AR and 8% at HR. In contrast, four surveys raised those Ψ estimates to 79% and 51%, respectively. This suggests that occupancy may be underestimated in 23% of surveyed pools at AR and 43% at HR if only surveyed once during the season.

## DISCUSSION

Our study demonstrates that imperfect detection is prevalent and highly variable in plants and lichens. While taxa with cryptic traits, such as annuals with seed banks or perennial geophytes with dormant life stages, have lower detectability on average, *p* varies substantially among taxa and studies. We found a relatively high *p* in a rare, annual plant, and yet even so, failing to consider detectability decreased the accuracy of occupancy models. Occupancy models for snorkelwort revealed that Ψ is shaped by soil depth, pool area, and site, while competitive vegetation can either increase or decrease *p* depending on the year. We estimate that a minimum of two repeated surveys within a season is sufficient for 95% confidence in detection and show that increasing survey effort increases the proportion of pools estimated to have high Ψ, suggesting single survey approaches underestimate the true Ψ of snorkelwort.

### Plants are detected imperfectly, and to varying degrees

Our review agrees with previous reviews, showing plants are detected imperfectly most of the time but at lower probabilities than found in some previous studies. We found that the mean *p* of 1427 plant and lichen taxa, representing many life history traits, was 0.46 (Figure 3A) which is much lower than the reported mean *p* ranging from 0.77 to 0.88 from Chen et al. (2013) for a range of grasses, forbs, and trees. Our distribution of *p* aligns more with Perret et al. (2023) who found a mean *p* of 0.44 (ranging from 0.11 to 0.82) for 30 herbaceous plant species with varying levels of conspicuousness. In our case study, we found that snorkelwort has a high detection probability, *p*, of 0.87. This is greater than the reported *p* estimates for 94% of the taxa from our literature review. However, even with a high *p*, in none of the repeat surveys did we detect snorkelwort present in 100% of the pools where it had been detected at least once in the season. Indeed, our estimate of detection for snorkelwort is likely to be a maximum estimate, since all surveys in our study were conducted by a single, experienced surveyor focused on this single species.

### Cryptic traits influence detection probability, mostly for annuals and geophytes, with exceptions

We found strong support that cryptic state and type influence *p* on average. Annual plants and deciduous geophytes had significantly lower *p* than non‐cryptic taxa, and among perennial non‐geophytes, deciduous plants have lower *p* than those that are evergreen. This effect is striking given that field surveys are intentionally conducted during highly conspicuous periods, like surveying in times of conspicuous leaf‐out, which should buffer negative effects of crypsis. By explicitly categorizing cryptic states, this study expands on previous research testing drivers of detectability, which generally find that a plant's morphology and conspicuousness impact its detectability (Dennett & Nielsen, 2018; Perret et al., 2023; van Strien et al., 2022). For example, Chen et al. (2013) found that when comparing the detectability between life forms, grasses had lower *p* than trees or shrubs, likely due to the relatively inconspicuous nature of grasses.

While our results support a general decrease in *p* for cryptic taxa, these traits explain remarkably little of the substantial variation in *p* across taxa and studies. Indeed, we found that certain annuals, deciduous geophytes, and deciduous non‐geophytic perennials exhibited some of the highest *p* (Begley‐Miller et al., 2019; Chen et al., 2013; Kéry et al., 2006; van Strien et al., 2022), including snorkelwort in this study. This suggests other taxon‐ or study‐specific effects play an important role in driving detection. For example, bright green snorkelwort is one of the first species to germinate in the season and contrasts visually with its immediate brown and gray environment, perhaps contributing to its high *p*. It also suggests that it may be difficult to anticipate the detectability of particular taxa without an explicit assessment of detection via repeat surveys.

### Imperfect detection and cryptic states in estimates of plant occupancy

There remains a paucity of papers that incorporate detection probabilities into estimates of plant occupancy. While our review focused only on papers using detection probabilities in plant/lichen systems, and not on how often occupancy is estimated, the 37 included papers represent a small fraction of such studies. We found that few of the studies we reviewed (*N* = 9, 24%) directly account for cryptic states in their modeling or survey design despite 75% of the studies including taxa with cryptic states. Of the studies that have accounted for cryptic states in real populations, they consistently found that cryptic states decrease species detection and can reduce overall estimates of population dynamics (Alexander et al., 2009; Lauriault & Wiersma, 2019).

Repeat surveys may not suffice to capture cryptic states as they can be inherently unobservable for an entire season, regardless of increased survey effort. Much of the existing literature addressing the role of cryptic states in detection and occupancy estimates has focused on developing sophisticated models using simulated datasets, limiting application to real systems (Lamy et al., 2013; Le Coz et al., 2019; Louvet et al., 2021). Dormant seed banks may be particularly consequential in multiyear occupancy studies which could falsely categorize a patch transition from absent to present as a colonization event if indeed the species was dormant (Manna et al., 2017). More studies are needed to address the role of cryptic traits at large, in estimates of both Ψ and *p* in real systems.

### Lessons from snorkelwort case study: Applications for a rare plant of conservation concern

Our case study revealed variation in *p* both between years and throughout the season, highlighting the risk of relying on a single annual survey. We suspect snorkelwort's cryptic seed bank and ephemeral life cycle to be the main cause of imperfect detection. For example, the species may senesce during sustained dry periods and be visually undetectable. Interestingly, we found that *p* decreased with greater competitive vegetation cover in 2023 and 2024 but increased with vegetative cover in 2025. This could reflect opposing effects of vegetative cover, as a visual impediment, a factor reducing snorkelwort abundance, or as an indicator of greater pool water holding capacity. Regardless of the drivers of snorkelwort detection, this case study emphasizes the value of repeat surveys, capturing scenarios where an ephemeral species is present but missed in a single survey. Indeed, the variation in occupancy estimates among surveys within a year is comparable to the variation in estimated occupancy among years, illustrating how imperfect detection in single surveys can confound observed trends over time. While resource constraints often limit monitoring, our results suggest that a minimum of two repeated surveys achieves a 95% confidence in detectability.

Snorkelwort occupancy varies among sites and is strongly dictated by pool characteristics. We identified the highest Ψ in larger pools and 3–5‐mm soil depths. While past work notes broader soil depth variation (up to 60 cm) shapes vegetation communities in granite outcrop pools (Burbanck & Phillips, 1983), our findings show the critical role of millimeter‐scale edaphic conditions for specialist endemics. AR had consistently higher Ψ and *p* than HR, which aligns with AR supporting the largest number of *G. amphiantha* populations, suggesting these metrics may reflect stronger, more resilient populations across the landscape. While this study did not directly test regional effects on population performance, characterizing microhabitat drivers of local occurrence offers essential insight into large‐scale population dynamics, highlighting the need for independent site management due to differing recovery needs (Mayberry & Elle, 2010). Past management for the species has included creating and deepening pools, directly manipulating soil depths and pool areas (Wyngaarden & Moffett, 2024); this study provides strong evidence that these factors directly contribute to pool occupancy. In conclusion, this case study provides evidence‐based targets for both monitoring efforts and habitat requirements for a rare species.

Incorporating estimates of *p* into Ψ estimates remains underutilized in plant systems and appears especially important for cryptic and/or rare taxa. Methods of incorporating specific cryptic states remain a growing frontier in the application of the detection probabilities for plants and should be considered when designing sampling and modeling methods. Without accounting for imperfect detection, harder‐to‐detect, or cryptic species, like snorkelwort, may be miscategorized as rarer than easier‐to‐detect species (Fisher, 2011). If left unaccounted for, this variation may be misattributed to larger trends of recovery or decline for the species, highlighting how small changes in estimated occupancy can have large conservation consequences (Garrard et al., 2015). The variation in *p*, and overall low detection on average, in plants highlights the need to further describe the detection probability of individual species, particularly for those species that are of high conservation concern and may suffer greater consequences from underestimates of occurrence.

## CONFLICT OF INTEREST STATEMENT

The authors declare no conflicts of interest.

## Supporting information


Appendix S1.

## Data Availability

Data (Wyngaarden, 2026) are available in Figshare at https://doi.org/10.6084/m9.figshare.30660926.v5. The species included in the case study is a federally protected species with sensitive locality data that have been anonymized.

## References

[ecy70525-bib-0046] Adamson, R. S. 1939. “The Classification of Life‐forms of Plants.” Botanical Review 5(10): 546–561. 10.1007/BF02868932.

[ecy70525-bib-0001] Akaike, H. 1974. “A New Look at the Statistical Model Identification.” IEEE Transactions on Automatic Control 19: 716–723.

[ecy70525-bib-0002] Alexander, H. M. , N. A. Slade , W. D. Kettle , G. L. Pittman , and A. W. Reed . 2009. “Detection, Survival Rates and Dynamics of a Cryptic Plant, Asclepias Meadii: Applications of Mark‐Recapture Models to Long‐Term Monitoring Studies.” Journal of Ecology 97: 267–276. 10.1111/j.1365-2745.2008.01468.x.

[ecy70525-bib-0003] Banner, K. M. , and M. D. Higgs . 2017. “Considerations for Assessing Model Averaging of Regression Coefficients.” Ecological Applications 27(1): 78–93. 10.1002/eap.1419. 27874997

[ecy70525-bib-0004] Baskin, J. M. , and C. C. Baskin . 1978. “The Seed Bank in a Population of an Endemic Plant Species and its Ecological Significance.” Biological Conservation 14: 125–130.

[ecy70525-bib-0005] Begley‐Miller, D. R. , D. R. Diefenbach , M. E. McDill , P. J. Drohan , C. S. Rosenberry , and E. H. Just Domoto . 2019. “Soil Chemistry, and Not Short‐Term (1‐2 Year) Deer Exclusion, Explains Understory Plant Occupancy in Forests Affected by Acid Deposition.” AoB Plants 11(5): plz044. 10.1093/aobpla/plz044. PMC6799995 31649810

[ecy70525-bib-0006] Beissinger, S. R. , S. M. Peterson , L. A. Hall , N. Van Schmidt , J. Tecklin , B. B. Risk , O. M. Richmond , T. J. Kovach , and A. M. Kilpatrick . 2022. “Stability of Patch‐Turnover Relationships under Equilibrium and Nonequilibrium Metapopulation Dynamics Driven by Biogeography.” Ecology Letters 25(11): 2372–2383. 10.1111/ele.14111. PMC9828715 36209497

[ecy70525-bib-0007] Bennett, J. R. , B. P. M. Edwards , J. N. Bergman , A. D. Binley , R. T. Buxton , D. E. L. Hanna , J. O. Hanson , et al. 2024. “How Ignoring Detection Probability Hurts Biodiversity Conservation.” Frontiers in Ecology and the Environment 22: e2782. 10.1002/fee.2782.

[ecy70525-bib-0008] Brooks, M. E. , K. Kristensen , K. J. van Benthem , A. Magnusson , C. W. Berg , A. Nielsen , H. J. Skaug , M. Mächler , and B. M. Bolker . 2017. “Modeling Zero‐Inflated Count Data with glmmTMB.” 10.1101/132753.

[ecy70525-bib-0009] Burbanck, M. P. , and D. L. Phillips . 1983. “Evidence of Plant Succession on Granite Outcrops of the Georgia Piedmont.” American Midland Naturalist 109(1): 94–104.

[ecy70525-bib-0010] Cade, B. S. 2015. “Model Averaging and Muddled Multimodel Inferences.” Ecology 96(9): 2370–2382. 10.1890/14-1639.1. 26594695

[ecy70525-bib-0011] Chen, G. , M. Kéry , M. Plattner , K. Ma , B. Gardner , and P. Zuidema . 2013. “Imperfect Detection Is the Rule Rather than the Exception in Plant Distribution Studies.” Journal of Ecology 101(1): 183–191. 10.1111/1365-2745.12021.

[ecy70525-bib-0012] Crisfield, V. E. , F. Guillaume Blanchet , C. Raudsepp‐Hearne , and D. Gravel . 2024. “How and why Species Are Rare: Towards an Understanding of the Ecological Causes of Rarity.” Ecography 2024: e07037. 10.1111/ecog.07037.

[ecy70525-bib-0013] Dennett, J. M. , and S. E. Nielsen . 2018. “Detectability of Species of Carex Varies with Abundance, Morphology, and Site Complexity.” Journal of Vegetation Science 30(2): 352–361. 10.1111/jvs.12713.

[ecy70525-bib-0014] Emry, D. J. , H. M. Alexander , and M. K. Tourtellot . 2011. “Modelling the Local Spread of Invasive Plants: Importance of Including Spatial Distribution and Detectability in Management Plans.” Journal of Applied Ecology 48(6): 1391–1400. 10.1111/j.1365-2664.2011.02050.x.

[ecy70525-bib-0015] Fisher, D. O. 2011. “Trajectories from Extinction: Where Are Missing Mammals Rediscovered?” Global Ecology and Biogeography 20(3): 415–425. 10.1111/j.1466-8238.2010.00624.x.

[ecy70525-bib-0016] Frings, D. M. , and L. J. Davenport . 2017. “Current Status of the Granite Pool Sprite, *Gratiola amphiantha* (Plantaginaceae), in Alabama.” Southeastern Naturalist 16(1): 59–69. 10.1656/058.016.0105.

[ecy70525-bib-0017] Garrard, G. E. , S. A. Bekessy , M. A. McCarthy , and B. A. Wintle . 2008. “When Have we Looked Hard Enough? A Novel Method for Setting Minimum Survey Effort Protocols for Flora Surveys.” Austral Ecology 33(8): 986–998. 10.1111/j.1442-9993.2008.01869.x.

[ecy70525-bib-0018] Garrard, G. E. , S. A. Bekessy , M. A. McCarthy , and B. A. Wintle . 2015. “Incorporating Detectability of Threatened Species into Environmental Impact Assessment.” Conservation Biology 29(1): 216–225. 10.1111/cobi.12351. 25155009

[ecy70525-bib-0019] Garrard, G. E. , M. A. McCarthy , N. S. G. Williams , S. A. Bekessy , B. A. Wintle , and S. Ramula . 2013. “A General Model of Detectability Using Species Traits.” Methods in Ecology and Evolution 4(1): 45–52. 10.1111/j.2041-210x.2012.00257.x.

[ecy70525-bib-0020] Goldstein, B. R. , A. G. Keller , K. L. Calhoun , K. J. Barker , F. Montealegre‐Mora , M. W. Serota , A. Van Scoyoc , P. Parker‐Shames , C. L. Andreozzi , and P. de Valpine . 2024. “How Do Ecologists Estimate Occupancy in Practice?” Ecography 2026: e07402. 10.1111/ecog.07402.

[ecy70525-bib-0021] Guillera‐Arroita, G. , J. J. Lahoz‐Monfort , D. I. MacKenzie , B. A. Wintle , and M. A. McCarthy . 2014. “Ignoring Imperfect Detection in Biological Surveys Is Dangerous: A Response to ‘Fitting and Interpreting Occupancy Models’.” PLoS One 9(7): e99571. 10.1371/journal.pone.0099571. PMC4116132 25075615

[ecy70525-bib-0022] Hauser, C. E. , K. M. Giljohann , M. A. McCarthy , G. E. Garrard , A. P. Robinson , N. S. G. Williams , and J. L. Moore . 2022. “A Field Experiment Characterizing Variable Detection Rates during Plant Surveys.” Conservation Biology 36(3): e13888. 10.1111/cobi.13888. PMC9303269 35098569

[ecy70525-bib-0023] Kellner, K. F. , and R. K. Swihart . 2014. “Accounting for Imperfect Detection in Ecology: A Quantitative Review.” PLoS One 9(10): e111436. 10.1371/journal.pone.0111436. PMC4214722 25356904

[ecy70525-bib-0024] Kéry, M. 2011. “Towards the Modelling of True Species Distributions.” Journal of Biogeography 38(4): 617–618. 10.1111/j.1365-2699.2011.02487.x.

[ecy70525-bib-0025] Kéry, M. , J. H. Spillmann , C. Truong , and R. Holderegger . 2006. “How Biased Are Estimates of Extinction Probability in Revisitation Studies?” Journal of Ecology 94(5): 980–986. 10.1111/j.1365-2745.2006.01151.x.

[ecy70525-bib-0026] Lamy, T. , O. Gimenez , J. P. Pointier , P. Jarne , and P. David . 2013. “Metapopulation Dynamics of Species with Cryptic Life Stages.” The American Naturalist 181(4): 479–491. 10.1086/669676. 23535613

[ecy70525-bib-0027] Lauriault, P. , and Y. F. Wiersma . 2019. “Reducing the Rate of False Absences of Cryptic Species in Inventory and Sampling Work.” The Bryologist 122(4): 578–585. 10.1639/0007-2745-122.4.xxx.

[ecy70525-bib-0028] Le Coz, S. , P. O. Cheptou , and N. Peyrard . 2019. “A Spatial Markovian Framework for Estimating Regional and Local Dynamics of Annual Plants with Dormancy.” Theoretical Population Biology 127: 120–132. 10.1016/j.tpb.2019.03.002. 31004605

[ecy70525-bib-0047] Lenth, R. , and J. Piaskowski . 2026. “emmeans: Estimated Marginal Means, aka Least‐Squares Means.” R package version 2.0.5. https://rvlenth.github.io/emmeans/.

[ecy70525-bib-0029] Louvet, A. , N. Machon , J. B. Mihoub , A. Robert , and R. Freckleton . 2021. “Detecting Seed Bank Influence on Plant Metapopulation Dynamics.” Methods in Ecology and Evolution 12(4): 655–664. 10.1111/2041-210x.13547.

[ecy70525-bib-0030] Lüdecke, D. , P. Waggoner , and D. Makowski . 2019. “insight: A Unified Interface to Access Information from Model Objects in R.” The Journal of Open Source Software 4(38): 1412. 10.21105/joss.01412.

[ecy70525-bib-0031] Lunsford, D. E. 1939. “Studies in the Lifecycle of *Amphianthus pusillus*.” MS thesis, Emory University.

[ecy70525-bib-0032] MacKenzie, D. I. , J. D. Nichols , G. B. Lachman , S. Droege , J. Andrew Royle , and C. A. Langtimm . 2002. “Estimating Site Occupancy Rates When Detection Probabilities Are Less than One.” Ecology 83(8): 2248–2255. 10.1890/0012-9658(2002)083[2248:Esorwd]2.0.Co;2.

[ecy70525-bib-0033] Manna, F. , R. Pradel , R. Choquet , H. Freville , and P. O. Cheptou . 2017. “Disentangling the Role of Seed Bank and Dispersal in Plant Metapopulation Dynamics Using Patch Occupancy Surveys.” Ecology 98(10): 2662–2672. 10.1002/ecy.1960. 28734092

[ecy70525-bib-0034] Mayberry, R. J. , and E. Elle . 2010. “Conservation of a Rare Plant Requires Different Methods in Different Habitats: Demographic Lessons from Actaea Elata.” Oecologia 164(4): 1121–1130. 10.1007/s00442-010-1809-8. 20963608

[ecy70525-bib-0035] Olea, P. P. , and P. Mateo‐Tomás . 2011. “Spatially Explicit Estimation of Occupancy, Detection Probability and Survey Effort Needed to Inform Conservation Planning.” Diversity and Distributions 17(4): 714–724. 10.1111/j.1472-4642.2011.00777.x.

[ecy70525-bib-0036] Perret, J. , A. Besnard , A. Charpentier , and G. Papuga . 2023. “Plants Stand Still but Hide: Imperfect and Heterogeneous Detection Is the Rule when Counting Plants.” Journal of Ecology 111(7): 1483–1496. 10.1111/1365-2745.14110.

[ecy70525-bib-0037] R Core Team . 2023. R: A Language and Environment for Statitical Computing. Version 4.3.2. Vienna: R Foundation for Statistical Computing.

[ecy70525-bib-0038] S. I. P. Ltd . 2025. “Procreate.” Version 5.4.10. Computer Software. Savage Interactive Pty Ltd.

[ecy70525-bib-0039] Schulz, T. , M. Saastamoinen , and J. Vanhatalo . 2025. “Model‐Based Variance Partitioning for Statistical Ecology.” Ecological Monographs 95(1): e1646. 10.1002/ecm.1646.

[ecy70525-bib-0040] Smithson, M. , and J. Verkuilen . 2006. “A Better Lemon Squeezer? Maximum‐Likelihood Regression with Beta‐Distributed Dependent Variables.” Psychological Methods 11(1): 54–71. 10.1037/1082-989X.11.1.54. 16594767

[ecy70525-bib-0041] Tucker, J. M. , K. M. Moriarty , M. M. Ellis , and J. D. Golding . 2021. “Effective Sampling Area Is a Major Driver of Power to Detect Long‐Term Trends in Multispecies Occupancy Monitoring.” Ecosphere 12(5): e03519. 10.1002/ecs2.3519.

[ecy70525-bib-0042] van Strien, A. J. , J. S. van Zweden , L. B. Sparrius , and B. Odé . 2022. “Improving Citizen Science Data for Long‐Term Monitoring of Plant Species in the Netherlands.” Biodiversity and Conservation 31(11): 2781–2796. 10.1007/s10531-022-02457-y.

[ecy70525-bib-0043] Weigelt, P. , C. König , and H. Kreft . 2019. “GIFT – A Global Inventory of Floras and Traits for Macroecology and Biogeography.” Journal of Biogeography 47(1): 16–43. 10.1111/jbi.13623.

[ecy70525-bib-0044] Wyngaarden, A. W. 2026. “Imperfect Detection Biases Occupancy Estimates in Plants: A Review and Case Study.” Figshare. Dataset. 10.6084/m9.figshare.30660926.v5.42775469

[ecy70525-bib-0045] Wyngaarden, A. W. , and J. M. Moffett . 2024. “Report on USFWS Invovlement in the Analysis and Evaluation of Granite Rock Outcrop Pool Enhancement/Creation Efforts.” https://iris.fws.gov/APPS/ServCat/DownloadFile/277222.

