# Supplementary material for: Imperfect detection biases occupancy estimates in plants: A review and case study
Source: Ecology. 2026 Sep 23;107(9):e70525. doi: 10.1002/ecy.70525 (PMC13598730; doi:10.1002/ecy.70525)
Supplement: Supplementary file 1 [file ECY-107-e70525-s001.pdf]

Appendix S1

Imperfect detection biases occupancy estimates in plants: A review and case study

Anna W. Wyngaarden, Megan L. DeMarche

Ecology

Literature review publication inclusion

We conducted a literature search on ISI's Web of Science on June 26, 2024 for publications between the year 2000 and the search date with the Topic "occupancy" OR "occupancy model" OR "occupancy modeling", and "plants" OR "floristic", and "detectability" OR "imperfect detection" OR "detection". This search returned 85 publications. We then screened those papers for those that 1) focused on plants/lichens as the study organism, 2) observed real organisms in the wild, and 3) incorporated imperfect detection into the estimates of species occurrence.

This reduced the list to 25 publications. We then added 12 additional papers that the authors were aware of that fit the criteria for inclusion but were not returned from the search. A total of 37 publications were reviewed for the literature review. Of the 37 publications, only 16 reported the p of taxa directly. From these 16, we compiled p estimates for 1,427 taxa, averaging across covariates or surveys when multiple estimates were provided.

Classifying life-form using Raunkiaer's system

We classified each taxon based on three traits – life cycle (annual or perennial), life-form, and deciduousness (deciduous or evergreen). Biennial taxa were considered perennial. We classified life-form following Raunkiaer's system which we obtained by calling "Life form 2" from Global Inventory of Floras and Traits (GIFT) database, using the R package, GIFT (Weigelt et al.,

2019). Raunkiaer's system classifies plants based on the location of their renewal buds, allowing us to distinguish taxa with unobservable, belowground states of dormancy (e.g. seed bank of therophytes, bulb-rhizome of geophytes) (Adamson, 1939). Specifically, life-forms were classified as a geophyte, phanerophyte, nanophanerophyte, hydrophyte, hemicryptophyte, helophyte, chamaephyte, or therophyte from the GIFT database. Missing or conflicting traits were resolved using alternative online databases (e.g. Missouri Botanical Garden's Plant Finder, The Royal Botanic Gardens Kew's Plants of the World Online, Minnesota Wildflowers) which are provided with all trait data (Wyngaarden 2026) in the document, LiteratureReview.csv, in the Figshare repository at:
https://doi.org/10.6084/m9.figshare.30660926.v5.

Mapping pools with suitable habitat

We mapped all unoccupied pools that were within five meters of an occupied pool and met suitable habitat criteria. Five meters is the average distance between occupied pools, a reasonable distance for dispersal. We calculated five meters as the average distance between occupied pools based on a separate study by the authors where occupied pools are mapped at 15 sites across the species' geographic range. Pools were mapped as georeferenced polygons with unique IDs and photos using an ArrowGold GPS (± 6cm precision) and Esri Field Maps.

We defined suitable snorkelwort habitat as having an obvious ability to retain water (e.g. defined pool edge, visible standing water at the time of survey), average soil depth of at least 2mm, and some visual amount of organic matter and bare soil.

Supplemental Figures and Tables

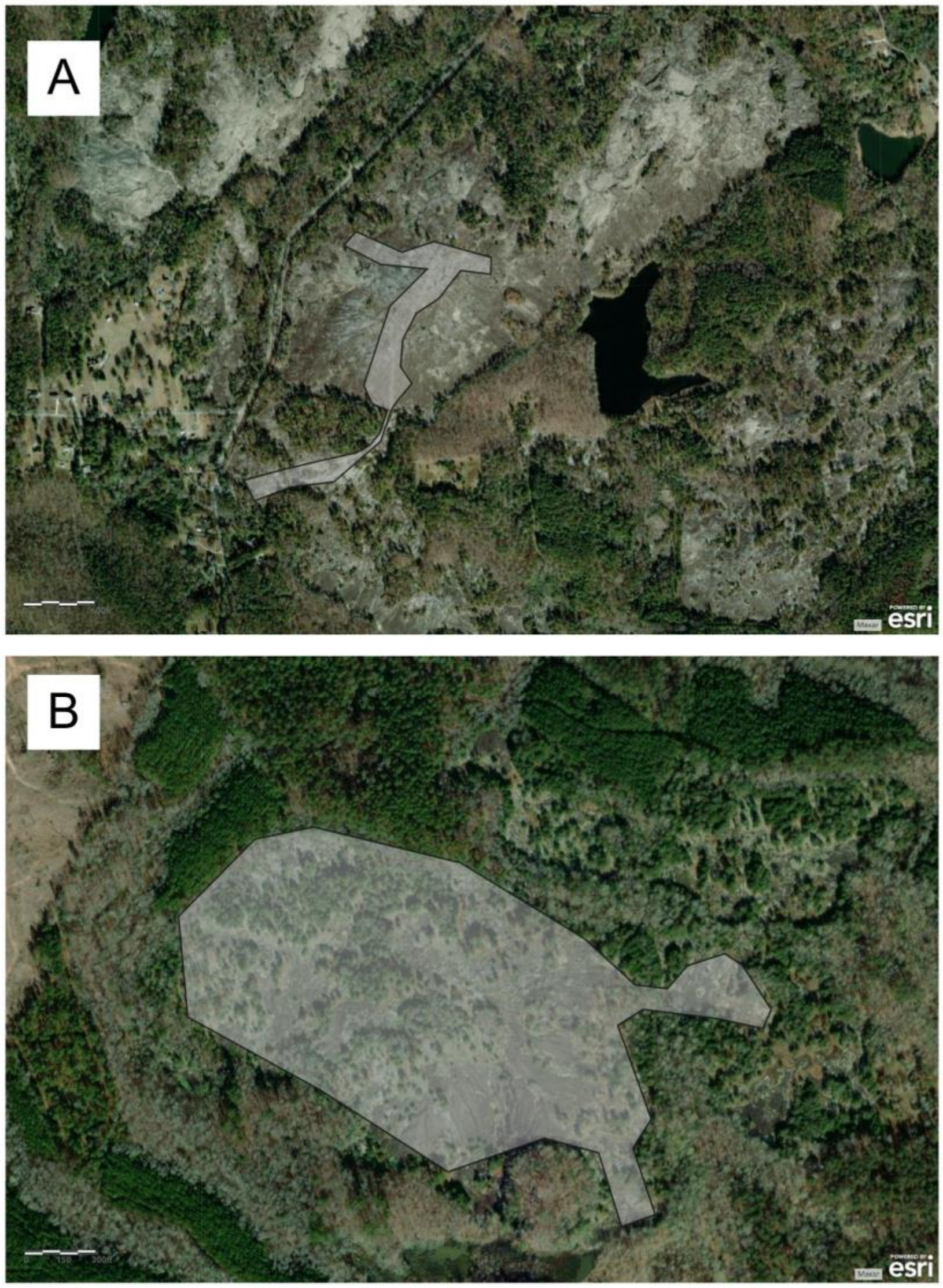

Figure S1. Study areas, the core portions of outcrop, are shown within shaded polygons at (A) Arabia (AR) and (B) Heggie's Rock (HR). Core portions contain the majority of contiguous suitable pool habitat at both sites. The full extent of each exposed granite outcrop is visible outside of the shaded polygons, where pools become more isolated. Map image is the intellectual property of Esri and is used herein under license. Copyright © 2026 Esri and its licensors. All rights reserved.

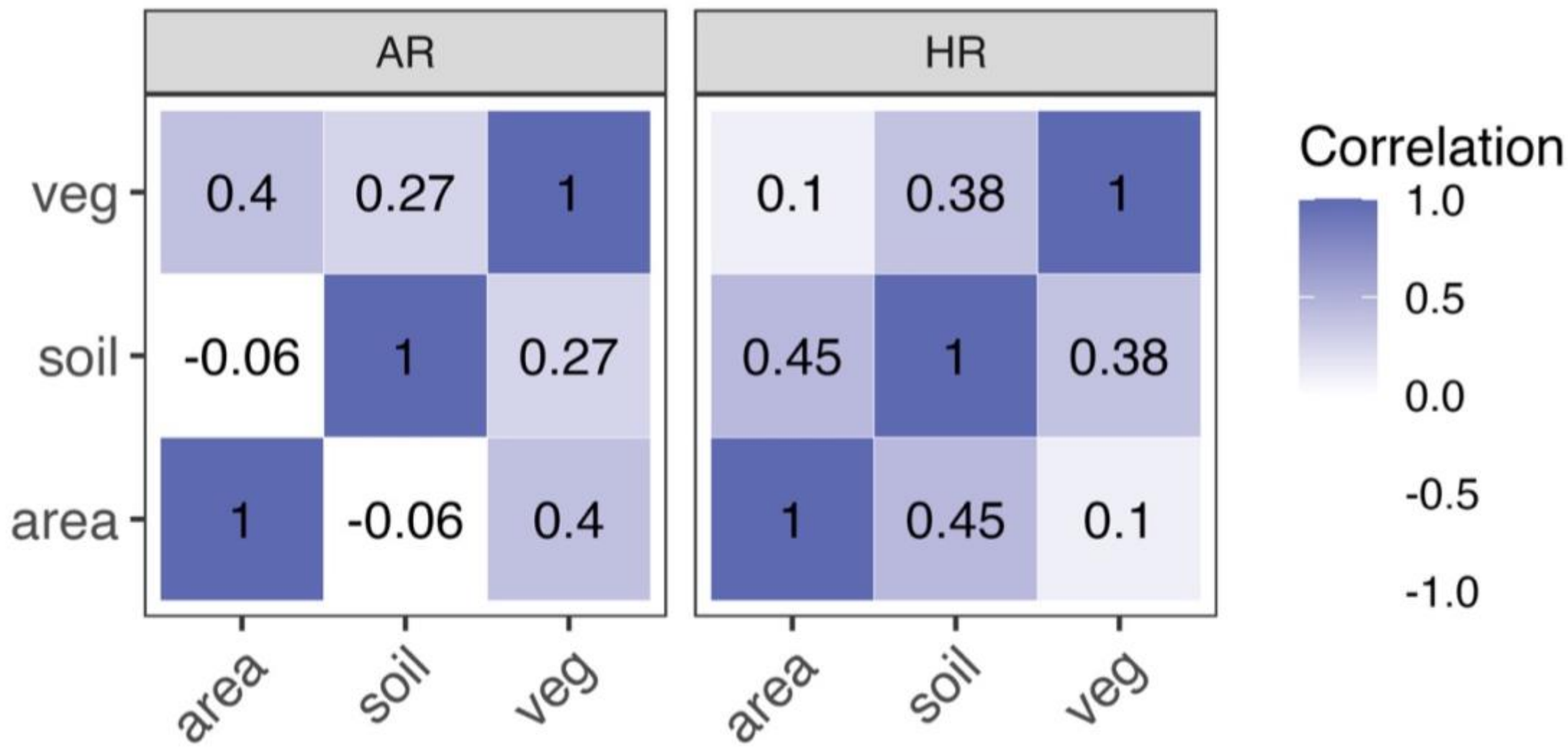

Figure S2. Correlation plots for pool habitat covariates at both sites, Arabia (AR) and Heggie's Rock (HR). Area is positively correlated with competing vegetation cover (veg) at both sites, and soil depth (soil) at HR. Soil depth and competing vegetation cover are positively correlated at both sites.

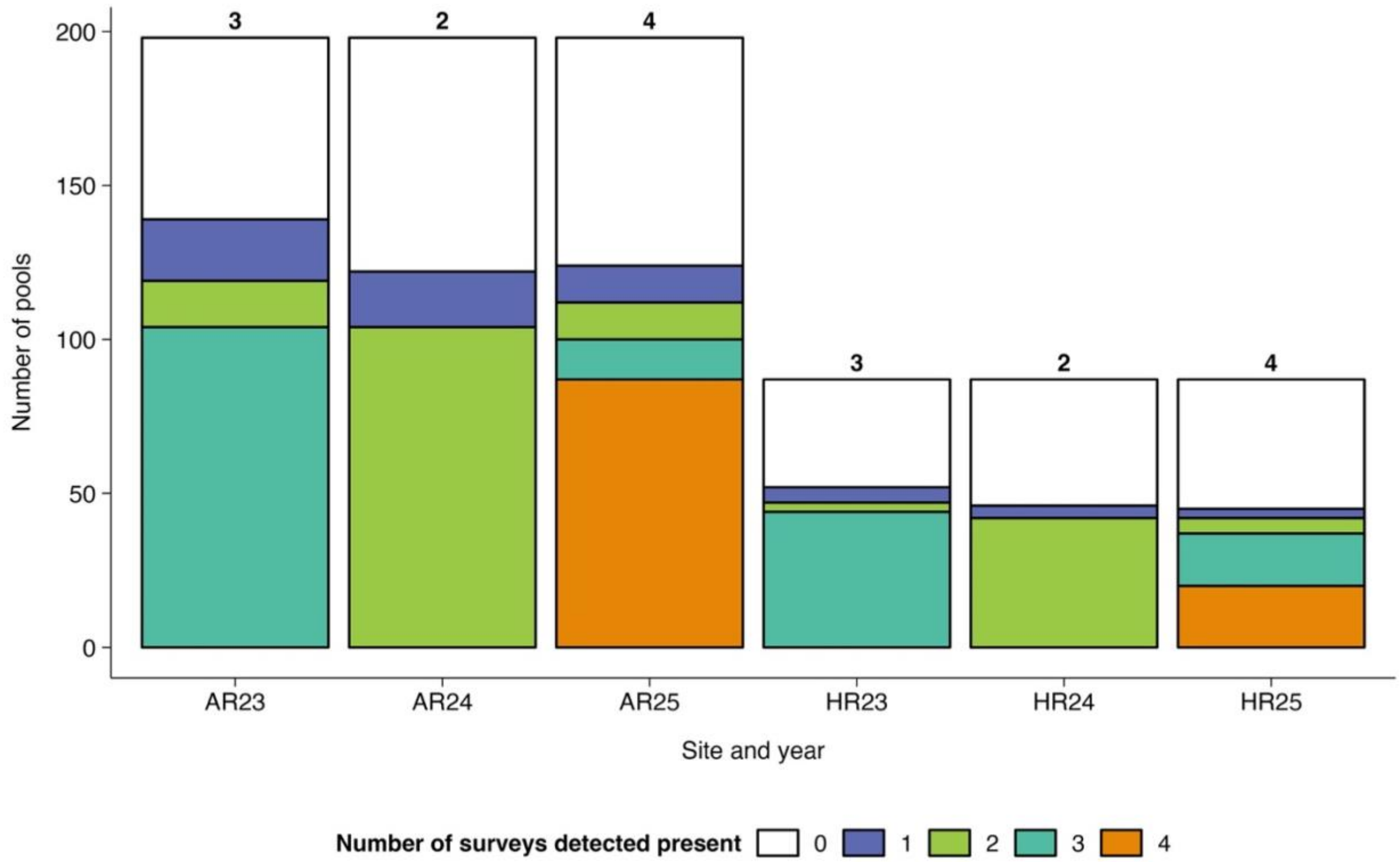

Figure S3. *Gratiola amphiantha* was never detected present in all pools that were occupied within a season. Colors show the number of times *G. amphiantha* was detected present in a pool out of the total number of surveys in a season (bolded numbers above bars) at both sites, Arabia (AR) and Heggie's Rock (HR). Some pools (N = 18) at AR were only surveyed twice in 2023 and were omitted from this graph.

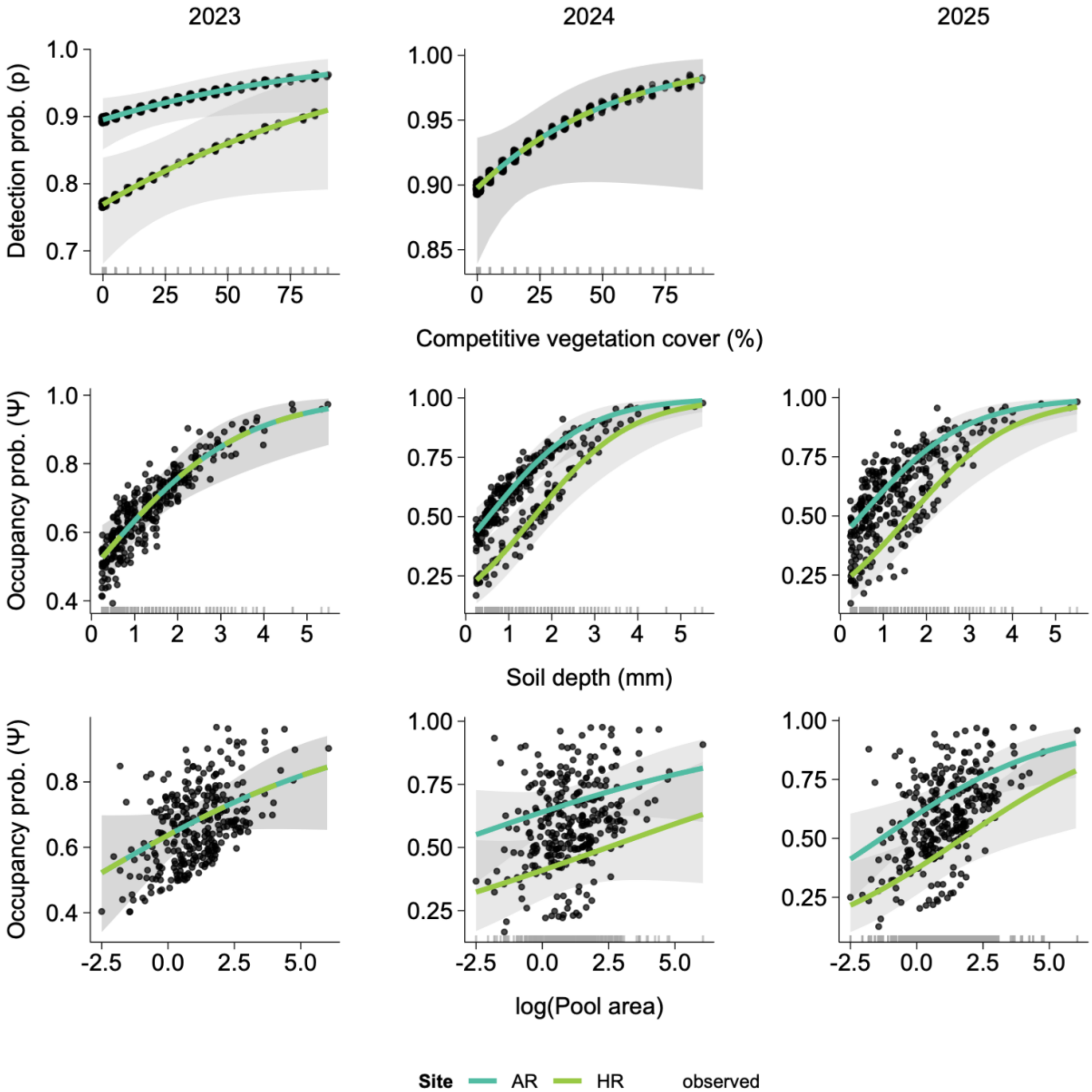

Figure S4. Detection (*p*) and occupancy (*Ψ*) probabilities vary across years and between sites. Detection probability varies with vegetation cover while occupancy probability increases with deeper soil and larger pools. Solid lines represent estimates from the top supported model for each year. Lines are colored by site (Arabia (AR), teal; Heggie's Rock (HR), green), and shaded regions indicate 95% CIs. Points represent pool estimates.

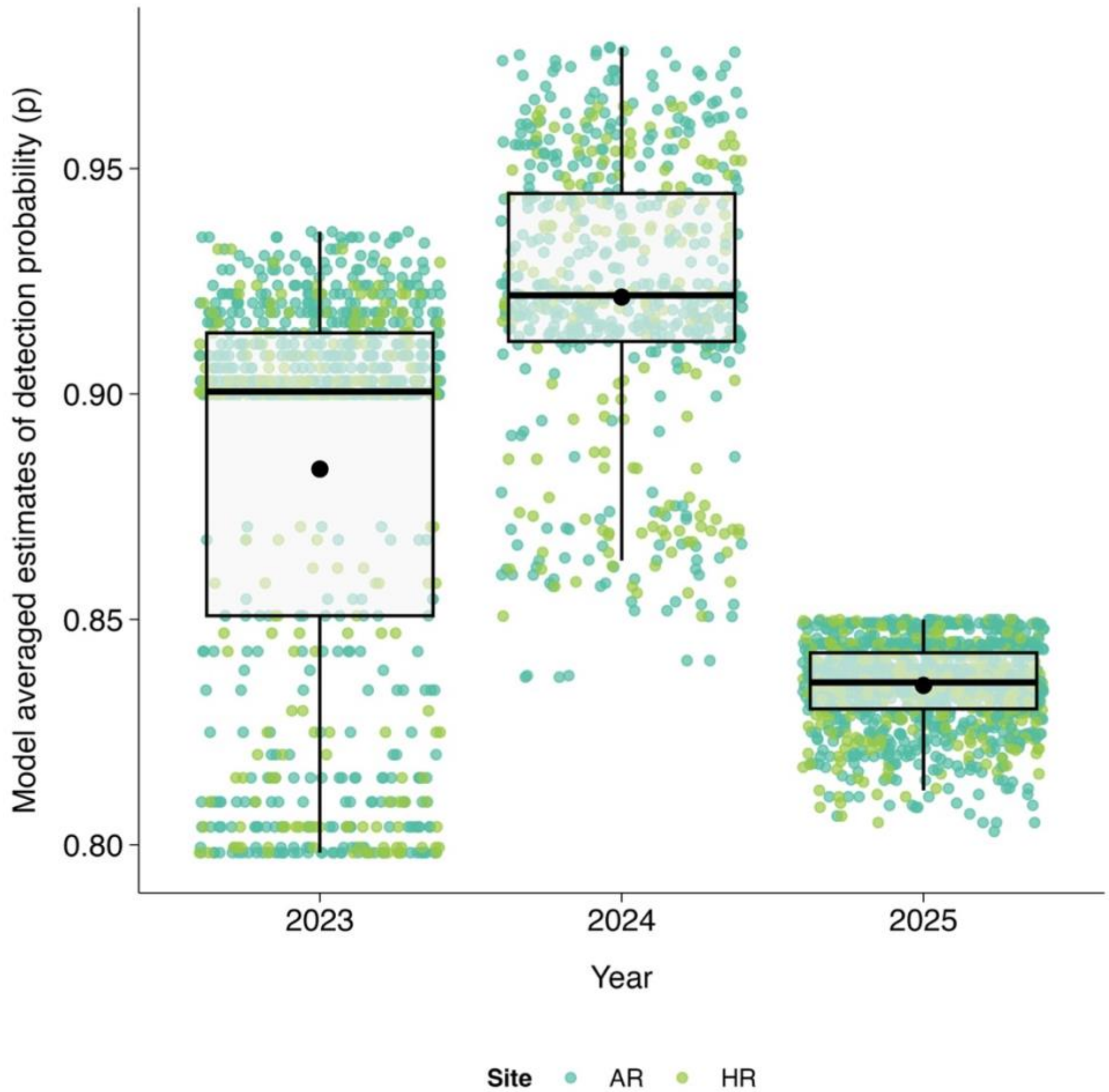

Figure S5. Snorkelwort is detected imperfectly ($p < 1$). Model averaged detection probability (p) estimates across survey years. Boxplots show the annual variation in detection probability for snorkelwort with black dots as annual means. Jittered points are survey-level detection estimates for pools, colored by site (Arabia (AR), teal; Heggie's Rock (HR), green). The average p for each site and year is based on model averaged estimates of p from models 2>ΔAIC of the top model.

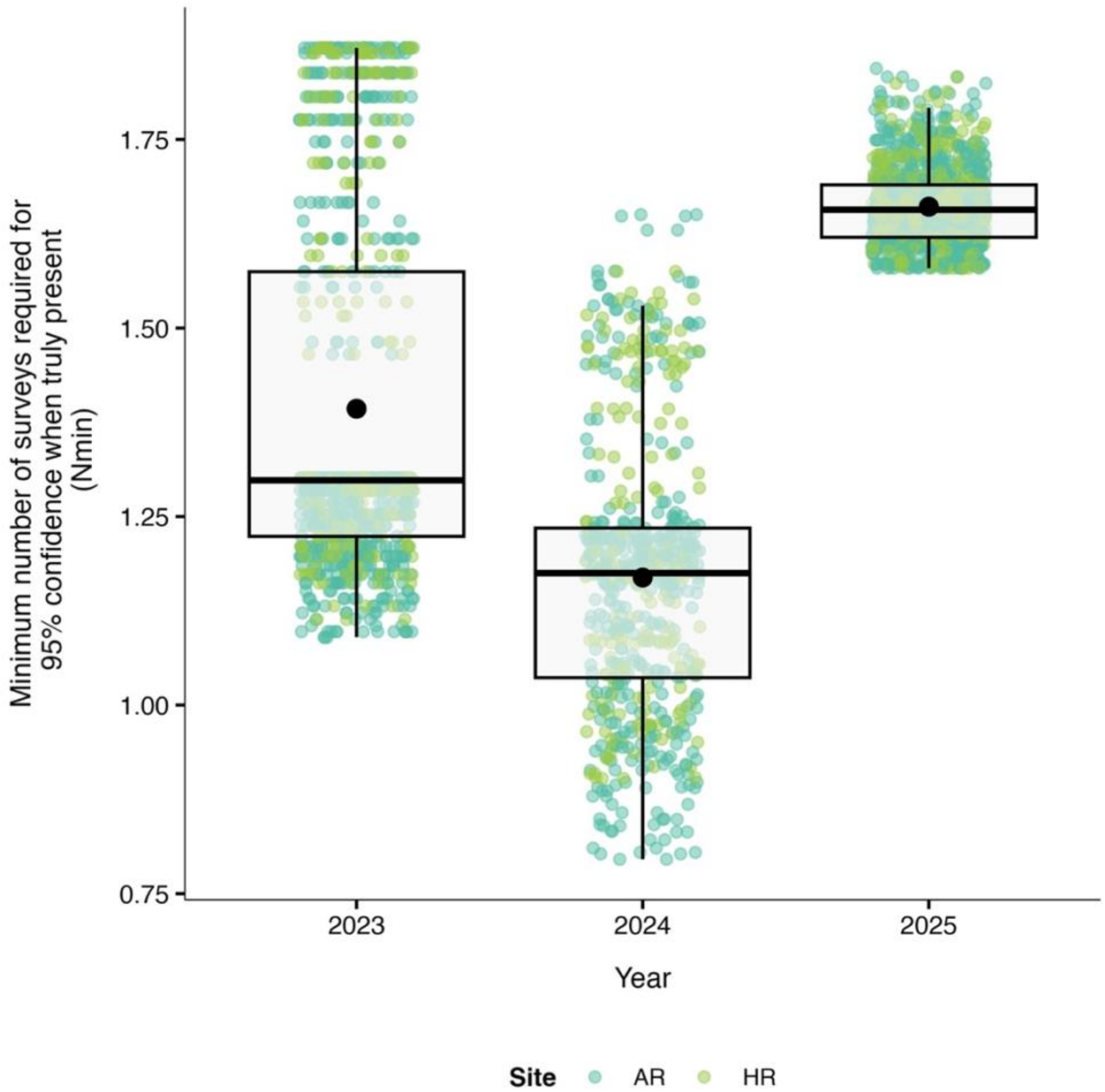

Figure S6. A minimum of 2 surveys is required to achieve 95% confidence that an occupied pool will be detected present. Boxplots show the distribution of N_{min} values for each year with black points as means. Colored points indicate N_{min} values of pools based on model averaged estimates of p and are colored by site (Arabia (AR), teal; Heggie's Rock (HR), green). N_{min} values less than 1 indicate p greater than 0.95.

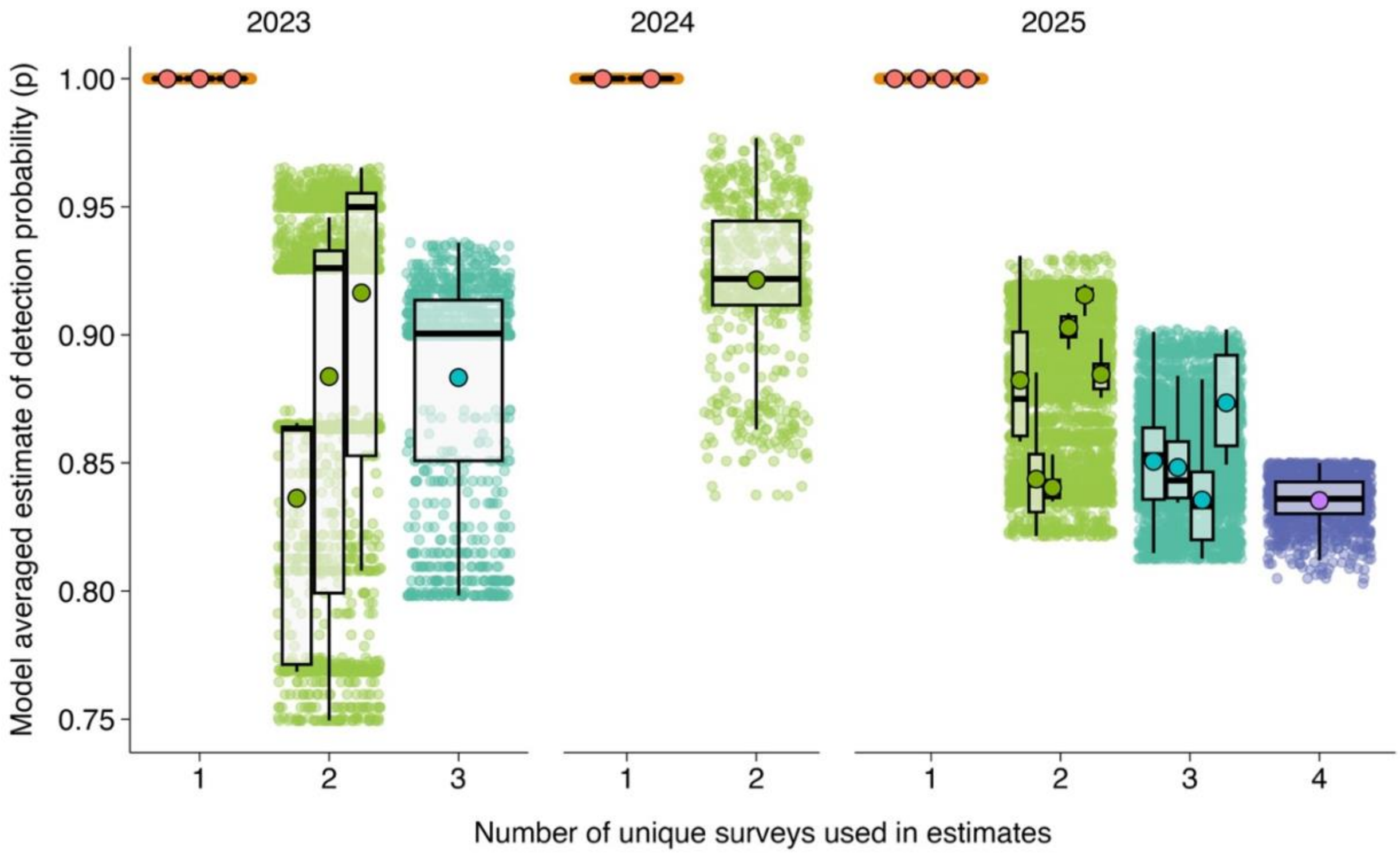

Figure S7. Model averaged detection probability (*p*) with varying survey effort. Boxplots represent different survey combinations used to generate the number of surveys from the whole season, 3 for 2023, 2 for 2024, and 4 for 2025. Estimates based on one survey included identical detections of a single survey. Colors are the number of unique surveys used to generate *p* estimates.

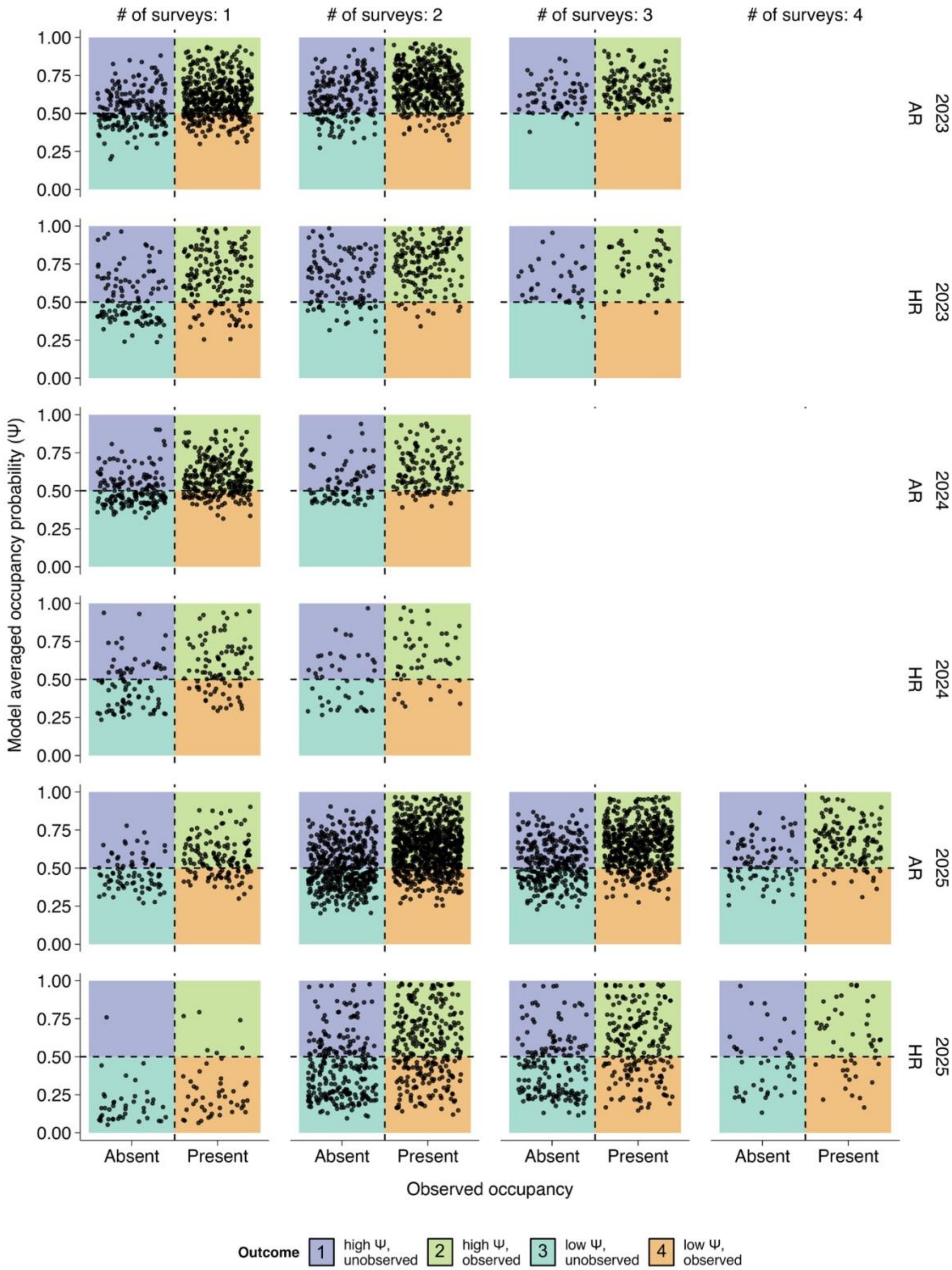

# of surveys: 1
# of surveys: 2
# of surveys: 3
# of surveys: 4
2023 AR
2023 HR
2024 AR
2024 HR
2025 AR
2025 HR
Model averaged occupancy probability (Ψ)
1.00
0.75
0.50
0.25
0.00
Absent
Present
Observed occupancy
Outcome
1 high Ψ, unobserved
2 high Ψ, observed
3 low Ψ, unobserved
4 low Ψ, observed

Figure S8 (previous page)**.** Comparison of observed detection to seasonal occupancy estimates (Ψ) with varying survey effort at both sites (Arabia (AR), Heggie's Rock (HR)). Pools estimated with high (≥0.50) or low (< 0.50) Ψ are compared to whether snorkelwort was observed absent every survey (0) or present at least once (1) during that season. Point shapes identify specific survey combinations. Quadrants are colored by their occupancy-observation outcome for each year and site (Outcome 1 (purple); Outcome 2 (green)*;* Outcome 3 (teal); Outcome 4 (orange)). High $\Psi \geq 0.50$ and low $\Psi < 0.50$. If imperfect detection affects model accuracy, increased survey effort should lead to more pools in these outcomes. Outcomes 1 and 4 indicate disagreement between model estimates and observations. Increased survey effort and improved detection models are expected to shift more pools into Outcome 1 because occupancy is likely underestimated due to missed detections. We expect fewer pools in outcome 4 with increased survey effort because fewer pools would be marked falsely absent in the season.

Table S1. Covariates considered as predictors for detection (p) and occupancy (Ψ) probability in our candidate model set.

**Variable**	**Definition**	**Justification**
Detectability (p)		
Doy	Day of year of survey with the first day set to November 1	p may vary throughout the growing season and this variable allows for within-season variation in p.
Area	Pool area (m^2)	p may decrease with larger pools because there is more area for false absences and increased survey area has been shown to decrease p. Alternatively, increased area may increase population abundance, which may increase p.
Veg	Percent cover of competing vegetation in surveyed pool area in 5% increments	High coverage of competing vegetation may reduce p, or may indicate pools retain water longer and increase p
Soil	Average soil depth of pool (mm)	Thinner soils dry out more quickly, increasing earlier senescence and reduce p.
Site	Site of survey	Site effects may influence p.
Occupancy (Ψ)		
Area	Pool area (m^2)	Larger pools are presumed to retain water longer, and are more likely to be colonized and retain populations, positively affecting Ψ.
Veg	Percent cover of competing vegetation in surveyed pool (5% increments)	High competitive vegetative cover reduces resource availability, negatively Ψ.
Soil	Average soil depth of pool (mm)	Deeper soils retain moisture and may support larger seed banks, positively affecting Ψ. Excessive depth may promote competition, limiting this relationship.
Site	Site of survey	Site effects may influence Ψ.

Table S2. Literature review model results. (A) Proportion of variance in model estimates from fixed effects, random effects, and residuals of the three literature review models. (B) Overall model results testing effect of cryptic state, type, and deciduous state on detection probability (*p*) from the literature review. Source paper was included as a random intercept in all models. (C) Estimated marginal means, EMM, on a logit scale for levels of each predictor. (D) Pairwise comparisons with a Tukey- adjustment for each model.

(A) Proportion of Variance

**Model**	**Component**	**Variance**	**Prop. of variance**
Cryptic state	Fixed effect	0.011	0.015
	Genus	0.157	0.207
	Publication	0.522	0.687
	Residual	0.070	0.910
Cryptic type	Fixed effect	0.015	0.021
	Genus	0.157	0.209
	Publication	0.508	0.678
	Residual	0.069	0.092
Deciduous state	Fixed effect	0.007	0.010
	Genus	0.213	0.318
	Publication	0.376	0.560
	Residual	0.075	0.112

(B) Overall Model Results

**Predictor**	χ^2	**df**	***p*-value**	$\mathbf{R^2}$ **(cond.;marg.)**
Cryptic state	12.613	1	0.0004	0.909; 0.015
Cryptic type	20.63	3	0.0001	0.908; 0.021
Deciduous state	5.522	1	0.0188	0.888; 0.010

(C) Estimated Marginal Means

**Predictor**	**Predictor level**	**EMM**	**SE**	**95% CI**
Cryptic state	no	0.846	0.231	0.394 – 1.30
	yes	0.635	0.230	0.185 – 1.09
Cryptic type	annual	0.554	0.235	0.0931 – 1.015
	deciduous	0.737	0.231	0.2852 – 1.190
	geophyte	0.513	0.240	0.0428 – 0.983
	none	0.859	0.229	0.4096 – 1.308
Deciduous state	deciduous	0.767	0.228	0.321 – 1.21
	evergreen	0.936	0.230	0.485 – 1.39

(D) Pairwise Comparisons (Tukey-adjusted)

**Predictor**	**Pairwise Comparison**	$\widehat{\beta}$	**SE**	**z.ratio**	***p*-value**
Cryptic state	no - yes	0.211	0.0595	3.552	0.0004
Cryptic type	annual - deciduous	-0.1833	0.0752	-2.438	0.0700
	annual - geophyte	0.0412	0.1167	0.353	0.9849
	annual - none	-0.3046	0.0774	-3.934	0.0005
	deciduous - geophyte	0.2245	0.1109	2.024	0.1789
	deciduous - none	-0.1213	0.0672	-1.805	0.2710
	geophyte - none	-0.3458	0.1095	-3.158	0.0086
Deciduous state	deciduous - evergreen	-0.169	0.0718	-2.350	0.0188

Table S3. Dates for each survey at both sites for each growing season.

**Site**	**Growing Season**	**Date of Survey**	**Days to Next Survey**
AR	2023	2/18/2023	27
		3/17/2023	14
		3/31/2023	--
	2024	2/2/2024	40
		3/13/2024	--
	2025	12/17/2024	29
		1/15/2025	34
		2/18/2025	28
		3/18/2025	--
HR	2023	2/14/2023	11
		2/25/2023	18
		3/14/2023	--
	2024	2/7/2024	30
		3/8/2024	--
	2025	12/12/2024	47
		1/28/2025	28
		2/25/2025	14
		3/11/2025	--

Table S4. List of 162 candidate models for occupancy (*Ψ*) and detectability (*p*) fit to each season's dataset.

#	**Candidate Model**
1	Ψ (1), p(1)
2	Ψ (1), p(area)
3	Ψ (1), p(veg)
4	Ψ (1), p(soil)
5	Ψ (1), p(site)
6	Ψ (1), p(doy)
7	Ψ (1), p(area + veg)
8	Ψ (1), p(area + soil)
9	Ψ (1), p(area + site)
10	Ψ (1), p(area + doy)
11	Ψ (1), p(veg + soil)
12	Ψ (1), p(veg + site)
13	Ψ (1), p(veg + doy)
14	Ψ (1), p(soil + site)
15	Ψ (1), p(soil + doy)
16	Ψ (1), p(site + doy)
17	Ψ (1), p(area + veg + soil)
18	Ψ (1), p(area + veg + site)
19	Ψ (1), p(area + veg + doy)
20	Ψ (1), p(area + soil + site)
21	Ψ (1), p(area + soil + doy)
22	Ψ (1), p(area + site + doy)
23	Ψ (1), p(veg + soil + site)
24	Ψ (1), p(veg + soil + doy)
25	Ψ (1), p(veg + site + doy)
26	Ψ (1), p(soil + site + doy)
27	Ψ (1), p(area + veg + soil + site)
28	Ψ (1), p(area + veg + soil + doy)
29	Ψ (1), p(area + veg + site + doy)
30	Ψ (1), p(area + soil + site + doy)
31	Ψ (1), p(veg + soil + site + doy)
32	Ψ (1), p(area + veg + soil + site + doy)
33	Ψ (area), p(1)
34	Ψ (area), p(veg)
35	Ψ (area), p(soil)
36	Ψ (area), p(site)
37	Ψ (area), p(doy)
38	Ψ (area), p(veg + soil)
39	Ψ (area), p(veg + site)

#	Candidate Model
40	Ψ (area), p(veg + doy)
41	Ψ (area), p(soil + site)
42	Ψ (area), p(soil + doy)
43	Ψ (area), p(site + doy)
44	Ψ (area), p(veg + soil + site)
45	Ψ (area), p(veg + soil + doy)
46	Ψ (area), p(veg + site + doy)
47	Ψ (area), p(soil + site + doy)
48	Ψ (area), p(veg + soil + site + doy)
49	Ψ (veg), p(1)
50	Ψ (veg), p(area)
51	Ψ (veg), p(soil)
52	Ψ (veg), p(site)
53	Ψ (veg), p(doy)
54	Ψ (veg), p(area + soil)
55	Ψ (veg), p(area + site)
56	Ψ (veg), p(area + doy)
57	Ψ (veg), p(soil + site)
58	Ψ (veg), p(soil + doy)
59	Ψ (veg), p(site + doy)
60	Ψ (veg), p(area + soil + site)
61	Ψ (veg), p(area + soil + doy)
62	Ψ (veg), p(area + site + doy)
63	Ψ (veg), p(soil + site + doy)
64	Ψ (veg), p(area + soil + site + doy)
65	Ψ (soil), p(1)
66	Ψ (soil), p(area)
67	Ψ (soil), p(veg)
68	Ψ (soil), p(site)
69	Ψ (soil), p(doy)
70	Ψ (soil), p(area + veg)
71	Ψ (soil), p(area + site)
72	Ψ (soil), p(area + doy)
73	Ψ (soil), p(veg + site)
74	Ψ (soil), p(veg + doy)
75	Ψ (soil), p(site + doy)
76	Ψ (soil), p(area + veg + site)
77	Ψ (soil), p(area + veg + doy)
78	Ψ (soil), p(area + site + doy)
79	Ψ (soil), p(veg + site + doy)
80	Ψ (soil), p(area + veg + site + doy)

#	**Candidate Model**
81	Ψ (site), p(1)
82	Ψ (site), p(area)
83	Ψ (site), p(veg)
84	Ψ (site), p(soil)
85	Ψ (site), p(doy)
86	Ψ (site), p(area + veg)
87	Ψ (site), p(area + soil)
88	Ψ (site), p(area + doy)
89	Ψ (site), p(veg + soil)
90	Ψ (site), p(veg + doy)
91	Ψ (site), p(soil + doy)
92	Ψ (site), p(area + veg + soil)
93	Ψ (site), p(area + veg + doy)
94	Ψ (site), p(area + soil + doy)
95	Ψ (site), p(veg + soil + doy)
96	Ψ (site), p(area + veg + soil + doy)
97	Ψ (area + veg), p(1)
98	Ψ (area + veg), p(soil)
99	Ψ (area + veg), p(site)
100	Ψ (area + veg), p(doy)
101	Ψ (area + veg), p(soil + site)
102	Ψ (area + veg), p(soil + doy)
103	Ψ (area + veg), p(site + doy)
104	Ψ (area + veg), p(soil + site + doy)
105	Ψ (area + soil), p(1)
106	Ψ (area + soil), p(veg)
107	Ψ (area + soil), p(site)
108	Ψ (area + soil), p(doy)
109	Ψ (area + soil), p(veg + site)
110	Ψ (area + soil), p(veg + doy)
111	Ψ (area + soil), p(site + doy)
112	Ψ (area + soil), p(veg + site + doy)
113	Ψ (area + site), p(1)
114	Ψ (area + site), p(veg)
115	Ψ (area + site), p(soil)
116	Ψ (area + site), p(doy)
117	Ψ (area + site), p(veg + soil)
118	Ψ (area + site), p(veg + doy)
119	Ψ (area + site), p(soil + doy)
120	Ψ (area + site), p(veg + soil + doy)

#	Candidate Model
121	Ψ (veg + soil), p(1)
122	Ψ (veg + soil), p(area)
123	Ψ (veg + soil), p(site)
124	Ψ (veg + soil), p(doy)
125	Ψ (veg + soil), p(area + site)
126	Ψ (veg + soil), p(area + doy)
127	Ψ (veg + soil), p(site + doy)
128	Ψ (veg + soil), p(area + site + doy)
129	Ψ (veg + site), p(1)
130	Ψ (veg + site), p(area)
131	Ψ (veg + site), p(soil)
132	Ψ (veg + site), p(doy)
133	Ψ (veg + site), p(area + soil)
134	Ψ (veg + site), p(area + doy)
135	Ψ (veg + site), p(soil + doy)
136	Ψ (veg + site), p(area + soil + doy)
137	Ψ (soil + site), p(1)
138	Ψ (soil + site), p(area)
139	Ψ (soil + site), p(veg)
140	Ψ (soil + site), p(doy)
141	Ψ (soil + site), p(area + veg)
142	Ψ (soil + site), p(area + doy)
143	Ψ (soil + site), p(veg + doy)
144	Ψ (soil + site), p(area + veg + doy)
145	Ψ (area + veg + soil), p(1)
146	Ψ (area + veg + soil), p(site)
147	Ψ (area + veg + soil), p(doy)
148	Ψ (area + veg + soil), p(site + doy)
149	Ψ (area + veg + site), p(1)
150	Ψ (area + veg + site), p(soil)
151	Ψ (area + veg + site), p(doy)
152	Ψ (area + veg + site), p(soil + doy)
153	Ψ (area + soil + site), p(1)
154	Ψ (area + soil + site), p(veg)
155	Ψ (area + soil + site), p(doy)
156	Ψ (area + soil + site), p(veg + doy)
157	Ψ (veg + soil + site), p(1)
158	Ψ (veg + soil + site), p(area)
159	Ψ (veg + soil + site), p(doy)

#	**Candidate Model**
160	Ψ (veg + soil + site), p(area + doy)
161	Ψ (area + veg + soil + site), p(1)
162	Ψ (area + veg + soil + site), p(doy)

Table S5. Model coefficients for occupancy probability (*Ψ*) and detection probability (*p*) of the top models (up to 2 ΔAIC from the top-ranked model).

Year	Model	Parameter	Covariate	$\hat{\beta}$	se
2023	Ψ(area + soil) p(veg + site)	Ψ	int	-0.24165	0.2486
			area	0.189007	0.1041
			soil	0.590986	0.1663
		p	int	2.143895	0.2048
			veg	0.012273	0.0063
			site	-0.941459	0.2617
	Ψ(area + veg + soil) p(site)	Ψ	int	-0.184543	0.2514
			area	0.257991	0.1101
			veg	-0.012356	0.0061
			soil	0.692566	0.1773
		p	int	2.369781	0.1754
			site	-0.924874	0.2605
	Ψ(soil) p(veg + site)	Ψ	int	-0.075047	0.2279
			soil	0.607313	0.164
		p	int	2.14427	0.2048
			veg	0.012267	0.0063
			site	-0.94126	0.2618
	Ψ(area + veg + soil + site) p(1)	Ψ	int	-0.076455	0.2568
			area	0.257565	0.1119
			veg	-0.013527	0.0062
			soil	0.852169	0.1913
			site	-0.953917	0.2977
		p	int	2.034771	0.1308
2024	Ψ(area + soil + site) p(veg)	Ψ	int	-0.631882	0.2559
			area	0.148849	0.103
			soil	0.888373	0.1845
			site	-0.941259	0.2972
		p	int	2.170194	0.2645
			veg	0.020331	0.0118
	Ψ(soil + site) p(area + veg)	Ψ	int	-0.494143	0.2335
			soil	0.898142	0.1796
			site	-0.940323	0.2953
		p	int	2.392213	0.3203
			area	-0.251746	0.1802

Year	Model	Parameter	Covariate	$\hat{\beta}$	se
			veg	0.027333	0.0133
	Ψ(soil + site) p(veg)	Ψ	int	-0.501124	0.2357
			soil	0.908348	0.1833
			site	-0.94587	0.2968
		p	int	2.168039	0.2651
			veg	0.020505	0.0118
	Ψ(area + soil) p(veg + site)	Ψ	int	-0.700411	0.2528
			area	0.150609	0.1013
			soil	0.728441	0.1687
		p	int	2.59176	0.322
			veg	0.020167	0.0118
			site	-1.352751	0.4095
	Ψ(soil) p(veg + site)	Ψ	int	-0.560996	0.2307
			soil	0.739046	0.1663
		p	int	2.589457	0.3224
			veg	0.020399	0.0118
			site	-1.353816	0.4095
	Ψ(soil) p(area + veg + site)	Ψ	int	-0.556605	0.229
			soil	0.732315	0.1639
		p	int	2.758646	0.3664
			area	-0.192325	0.1758
			veg	0.024879	0.013
			site	-1.314282	0.4153
	Ψ(area + soil + site) p(1)	Ψ	int	-0.647371	0.2596
			area	0.151028	0.1033
			soil	0.900518	0.1892
			site	-0.940132	0.2981
		p	int	2.495657	0.2177
	Ψ(soil + site) p(1)	Ψ	int	-0.513063	0.2385
			soil	0.918796	0.1874
			site	-0.944099	0.2975
		p	int	2.497969	0.2175
2025	Ψ(area + veg + soil + site) p(1)	Ψ	int	-0.714329	0.2555
			area	0.30501	0.1066
			soil	0.824114	0.1751
			site	-0.931893	0.2942
		p	int	1.626197	0.1009
	Ψ(area + soil + site) p(veg)	Ψ	int	-0.714329	0.2555

Year	Model	Parameter	Covariate	$\widehat{\beta}$	se
			area	0.30501	0.1066
			soil	0.824114	0.1751
			site	-0.931893	0.2942
		p	int	1.736083	0.1331
			veg	-0.00535	0.004
	Ψ(area + soil + site) p(doy)	Ψ	int	-0.714329	0.2555
			area	0.304994	0.1066
			soil	0.824043	0.1751
			site	-0.931852	0.2942
		p	int	1.943827	0.2879
			doy	-0.003397	0.0028
	Ψ(area + soil + site) p(veg + doy)	Ψ	int	-0.713611	0.2554
			area	0.304966	0.1066
			soil	0.823488	0.1749
			site	-0.931771	0.2942
		p	int	2.051234	0.3038
			veg	-0.005323	0.004
			doy	-0.003376	0.0028
	Ψ(area + veg + soil + site) p(1)	Ψ	int	-0.722024	0.2579
			area	0.300449	0.1106
			veg	0.000933	0.0061
			soil	0.817428	0.1805
			site	-0.92992	0.2945
		p	int	1.62618	0.1009

Table S6. The mean detection probability (*p*) estimates for varying numbers (*k* = 1-4) and combinations of detection surveys (S1-S4) across years. Note that when using a single survey (*k*=1), we duplicated the same survey twice in the dataset to enable occupancy models to run; this is akin to assuming perfect detection. Estimates are model-averaged from the top supported occupancy models using AIC weights for each year's full dataset.

k	**Detection surveys used**	**Mean detection probability**
1	$S1_{2023}$	1.00
1	$S2_{2023}$	1.00
1	$S3_{2023}$	1.00
2	$S1_{2023}$, $S2_{2023}$	0.836
2	$S1_{2023}$, $S3_{2023}$	0.884
2	$S2_{2023}$, $S3_{2023}$	0.916
3	$S1_{2023}$, $S2_{2023}$, $S3_{2023}$	0.883
1	$S1_{2023}$	1.00
1	$S2_{2024}$	1.00
2	$S1_{2024}$, $S2_{2024}$	0.921
1	$S1_{2025}$	1.00
1	$S2_{2025}$	1.00
1	$S3_{2025}$	1.00
1	$S4_{2025}$	1.00
2	$S1_{2025}$, $S2_{2025}$	0.882
2	$S1_{2025}$, $S3_{2025}$	0.844
2	$S1_{2025}$, $S4_{2025}$	0.840
2	$S2_{2025}$, $S3_{2025}$	0.903
2	$S2_{2025}$, $S4_{2025}$	0.916
2	$S3_{2025}$, $S4_{2025}$	0.885
3	$S1_{2025}$, $S2_{2025}$, $S3_{2025}$	0.851
3	$S1_{2025}$, $S2_{2025}$, $S4_{2025}$	0.848
3	$S1_{2025}$, $S3_{2025}$, $S4_{2025}$	0.836
3	$S2_{2025}$, $S3_{2025}$, $S4_{2025}$	0.873
4	$S1_{2025}$, $S2_{2025}$, $S3_{2025}$, $S4_{2025}$	0.835

References

Adamson, R. S. (1939). The classification of life-forms of plants. *Botanical Review*, *5*(10), 546-561.

Schulz, T., Saastamoinen, M., & Vanhatalo, J. (2025). Model-based variance partitioning for statistical ecology. *Ecological Monographs*, *95*(.). https://doi.org/10.1002/ecm.1646

Weigelt, P., König, C., & Kreft, H. (2019). GIFT – A Global Inventory of Floras and Traits for macroecology and biogeography. *Journal of Biogeography*, *47*(.), 16-43. https://doi.org/10.1111/jbi.13623

Wyngaarden, A. W. (2026). *Imperfect detection biases occupancy estimates in plants: a review and case study*. Figshare. Dataset. https://doi.org/https://doi.org/10.6084/m9.figshare.30660926.v5.
